# Novel PEPPSI-Type NHC Pd(II) Metallosurfactants on the Base of 1H-Imidazole-4,5-dicarboxylic Acid: Synthesis and Catalysis in Water–Organic Media

**DOI:** 10.3390/nano12224100

**Published:** 2022-11-21

**Authors:** Vladimir Burilov, Dmitriy Radaev, Elza Sultanova, Diana Mironova, Daria Duglav, Vladimir Evtugyn, Svetlana Solovieva, Igor Antipin

**Affiliations:** 1 Kazan Federal University, 18 Kremlevskaya St., 420008 Kazan, Russia; 2 Interdisciplinary Center for Analytical Microscopy, Kazan Federal University, 18 Kremlevskaya St., 420008 Kazan, Russia; 3 Arbuzov Institute of Organic and Physical Chemistry, FRC Kazan Scientific Center of RAS, 8 Arbuzov Str., 420008 Kazan, Russia

**Keywords:** PEPPSI complex, NHC, aggregation, *p*-nitrophenol reduction, Suzuki–Miyaura coupling

## Abstract

Carrying out organic reactions in water has attracted much attention. Catalytic reactions in water with metallosurfactants, which have both a metallocenter and the surface activity necessary for solubilizing hydrophobic reagents, are of great demand. Herein we proposed new approach to the synthesis of NHC PEPPSI metallosurfactants based on the sequential functionalization of imidazole 4,5-dicarboxylic acid with hydrophilic oligoethylene glycol and lipophilic alkyl fragments. Complexes of different lipophilicity were obtained, and their catalytic activity was studied in model reduction and Suzuki–Miyaura reactions. A comparison was made with the commercial PEPPSI-type catalytic systems designed by Organ. It was found that the reduction reaction in an aqueous solution of the metallosurfactant with the tetradecyl lipophilic fragment was three times more active than the commercially available PEPPSI complexes, which was associated with the formation of stable monodisperse aggregates detected by DLS and TEM.

## 1. Introduction

A classic problem in chemistry is the reaction between reactants of different solubilities. This is especially true for organic reactions carried out in water. This is also relevant for catalytic reactions, since bulk water-insoluble ligands are often used for metal complex compounds. Nevertheless, carrying out organic reactions in water [1,2,3] is relevant in the light of the concept of green chemistry [4] and, to some extent, mimics nature, which uses water as a medium for all its reactions. The recipe for eliminating the problem of two immiscible media is known—the use of micellar catalysis [5,6]. Traditional surfactants consist of both a polar hydrophilic and hydrophobic part. The introduction of a metal center into the structure of a surfactant makes it possible to obtain a qualitatively new type of structure endowed with catalytic properties in addition to surface activity. These structures are known as metallosurfactants [7,8,9]. Interest in such systems has grown significantly in the last decade due to the possibility of using metallosurfactants both for catalysis [10] and as precursors for obtaining structured nanoparticles [11], as templates for mesoporous nanomaterials [12], and even in medicine [13]. Since there are several examples of phosphine–Pd metallosurfactants [14,15] or imine-chelating Pd metallosurfactants [16,17], of particular interest are metallosurfactant systems based on N-heterocyclic carbenes (NHC) due to their unique catalytic activity as well as their resistance to moisture and atmospheric oxygen [18,19]. In the last few years, examples of metallosurfactants with the polar fragment in the N position of the imidazolium ring have appeared in the literature. For example, complexes of Pd(II), Ag(I), and Au(I) were synthesized with an oligoethylene glycol polar fragment [20,21,22,23], a series of PEPPSI-type (pyridine-enhanced precatalyst preparation stabilization and initiation) Pd(II) complexes with a carbohydrate residue in the N-position were obtained [24], and amphiphilic bis-NHC chelating complexes of Cu(I) anf Fe(II) were reported [25].

**Scheme 1:**
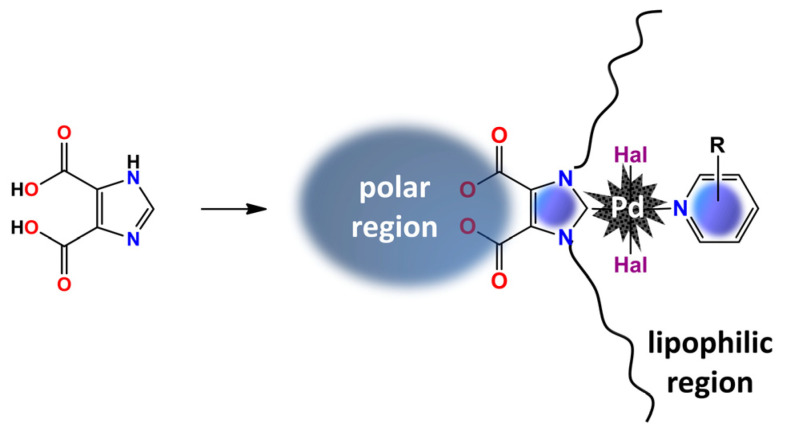
Metallosurfactant on a 1H-imidazole-4,5-dicarboxylic acid platform.

In this work, we proposed new approach to the synthesis of gemini-like NHC metallosurfactans based on the stepwise functionalization of 1H-imidazole-4,5-dicarboxylic acid (Scheme 1). Due to the presence of multiple reaction centers, the structure of the final catalyst could be further tuned by introducing different polar groups at the C4 and C5 positions and different hydrophobic groups at the N-positions of the heterocycle core.

## 2. Materials and Methods

### 2.1. Characterisation Methods

^1^H and ^13^C NMR spectra as well as 2D ^1^H-^1^H NOESY were recorded on a Bruker Avance 400 Nanobay (Bruker Corporation, Billerica, MA, USA) device with signals from residual protons of CDCl_3_ or DMSO-d6 used as the internal standard. 

The melting points were measured using an OIptimelt MPA100 melting point apparatus (Stanford Research Systems, Sunnyvale, CA, USA). 

IR spectra in KBr pellets were recorded on a Bruker Vector-22 spectrometer (Bruker Corporation, Billerica, MA, USA). 

High-resolution mass spectra with electrospray ionization (HRESI MS) were obtained on an Agilent iFunnel 6550 Q-TOF LC/MS (Agilent Technologies, Santa Clara, CA, USA) device in the positive mode. The following parameters were used: nitrogen carrier gas, temperature 300 °C, carrier flow rate 12 L·min^−1^, nebulizer pressure 275 kPa, funnel voltage 3500 V, capillary voltage 500 V, total ion current recording mode, 100–3000 m/z mass range, and scanning speed 7 spectra·s^−1^.

### 2.2. Reagents

Chemicals were purchased from commercial suppliers and used as received. 1H-imidazole-4,5-dicarboxylic acid was obtained by benzimidazole oxidation [26]. Solvents were purified according to standard procedures. Substance purity and the process of reaction were monitored by TLC on Merck UV 254 plates and visualized by exposure to UV with a VL-6.LC lamp (Vilber, Marne-la-Vallée, France). 

Synthesis of 4,5-bis[2-(2-(2-methoxyethoxy)ethoxy)ethoxycarbonyl]imidazole (**1**).

In a round-bottomed flask, thionyl chloride (3.4 mL, 46.7 mmol) was added dropwise to a suspension of imidazole-4,5-dicarboxylic acid (1.2 g, 7.8 mmol) in toluene (15 mL) in a cold water bath followed by the addition of catalytic amount of DMF (0.3 mL). The mixture was stirred at 50 °C for 20 h. Then, 5 mL of DCM was added, the solvents were evaporated under reduced pressure and this procedure was repeated twice. A total of 35 mL of DCM was added to the yellow residue, and the solution was cooled in an ice bath. A mixture of 2-(2-(2-methoxyethoxy)ethoxy)ethoxyethanol (3.7 mL, 23.4 mmol) and triethylamine (2.2 mL, 15.6 mmol) in 5 mL of DCM was added to the solution dropwise. The mixture was stirred at 0 °C for 1 h and then at room temperature for 17 h. The solvent was evaporated under reduced pressure, and the brown liquid was diluted with 30 mL of acetone. The white precipitate was filtered, the filtrate was evaporated, and the brown residue oil was diluted with 20 mL of water and extracted with hexane (3 × 20 mL) and Et_2_O (2 × 20 mL). The water layer was evaporated, and the product was obtained as a yellow oil. The yield was 2.2 g (63%), and the TLC R_f_ = 0.25 (CHCl_3_: MeOH, 10:1).

^1^H NMR (400 MHz, CDCl_3_, 25 °C): δ, ppm: 3.37 (s, 6H, O-CH_3_), 3.53–3.58 (m, 4H, CH_3_-O-CH_2_), 3.61–3.70 (m, 12 H, O-CH_2_), 3.80 (brt, J = 4.8 Hz, 4H, C(O)-O-CH_2_-CH_2_), 4.48 (brt, J = 4.4 Hz, 4H, C(O)-O-CH_2_), 7.85 (s, 1H, Imd CH).

^13^C NMR (100.6 MHz, CDCl_3_, 25 °C): δ, ppm: 160.12, 137.78, 130.23, 71.50, 70.18, 70.11, 69.99, 68.59, 63.88, 58.57

IR (KBr) ν_max_ cm^−1^: 2884 (C-H), 1727 (C=O), 1546 (Imd), 1453 (CH_3_), 1293 (C(O)-O), 1106 (C-O-C)

HR ESI MS (m/z) [M+Na]^+^ calcd. for C_19_H_32_N_2_NaO_10_^1+^ = 471.1949, found 471.1952, [M+H]^+^ calcd. for C_19_H_33_N_2_O_10_^1+^ = 449.2129, found 449.2131, [M-H+2Na]^1+^ calcd. for C_19_H_31_Na_2_N_2_O_10_^1+^ = 493.1768, found 493.1769, [M+Na]^1+^ calcd. for C_19_H_32_N_2_KO_10_^1+^ = 487.1694, found 487.1684.

Synthesis of 4,5-bis[2-(2-(2-methoxyethoxy)ethoxy)ethoxycarbonyl]-1-methylimidazole (**2a**) and 4,5-bis[2-(2-(2-methoxyethoxy)ethoxy)ethoxycarbonyl]-1-tetradecylimidazole (**2b**).

A total of 0.4 g (0.9 mmol) of 4,5-bis[2-(2-(2-methoxyethoxy)ethoxy)ethoxycarbonyl]imidazole 1, 0.06 mL (1 mmol) of methyl iodide or 0.40 mL (1.4 mmol) of tetradecyl bromide (for **2b**), 0.13 g (0.9 mmol) of potassium carbonate, and 7 mL of acetone were added to a GlassChem reactor. The mixture was stirred at 40 °C for 1 h (24 h for **2b**). Then, the solvent was evaporated under reduced pressure, the residue was diluted with DCM, and the inorganic precipitate was filtered.

For **2a,** the filtrate was evaporated, and the residue was extracted from water (10 mL) with hexane (3 × 10 mL) and Et_2_O (3× 10 mL). The water layer was evaporated, and the product was obtained as a pale-yellow liquid. The yield was 0.35 g (85%), and the TLC R_f_ = 0.34 (CHCl_3_: MeOH, 10:1).

For **2b,** the filtrate was evaporated, and the residue was purified by column chromatography using chloroform–acetone (5:1) as the eluent. The product was obtained as a pale-yellow oil. The yield was 0.33 g (57%) and the TLC R_f_ = 0.58 (CHCl_3_: MeOH, 10:1).

**2a** ^1^H NMR (400 MHz, CDCl_3_, 25 °C): δ, ppm: 3.37 (s, 3H, OCH_3_), 3.38 (s, 3H, OCH_3_), 3.51–3.57 (m, 4H, CH_2_-O-CH_3_), 3.61–3.71 (m, 12H, OCH_2_), 3.78–3.83 (m, 4H, COOCH_2_-CH_2_), 3.84 (s, 3H, N-CH_3_), 7.51 (s, 1H, Imd CH).

^13^C NMR (100.6 MHz, CDCl_3_, 25 °C): δ, ppm: 162.18, 159.62, 140.00, 136.98, 125.44, 72.46, 71.79, 71.77, 70.49, 70.47, 70.45, 70.41, 70.34, 70.18, 68.76, 68.59, 64.48, 64.13, 61.51, 58.91

IR (KBr) ν_max_ cm^−1^: 2884 (C-H), 1722 (C=O), 1542 (Imd), 1435 (CH**_3_**) 1223 (C(O)-O), 1108 (C-O-C)

HR ESI MS (m/z) [M+H]^1+^ calcd. for C_20_H_35_N_2_O_10_^1+^ = 463.2287, found 463.2289; [M+Na]^1+^ calcd. for C_20_H_34_N_2_NaO_10_^1+^ = 485.2106, found 485.2105.

**2b** ^1^H NMR (400 MHz, CDCl_3_, 25^o^C): δ, ppm: 0.88 (t, J = 6.6 Hz, 3H C_14_ CH_3_), 1.20–1.33 (m, 22H, C_14_ CH_2_), 1.71–1.80 (m, 2H, N-CH_2_-CH_2_), 3.37 (s, 6H, O-CH_3_), 3.52–3.55 (m, 4H, CH_3_-O-CH_2_), 3.61–3.70 (m, 12 H, OCH_2_), 3.77–3.83 (m, 4H, C(O)-O-CH_2_-CH_2_), 4.17 (t, J = 7.2 Hz, 2H, N-CH_2_), 4.44–4.49 (m, 4H, C(O)-O-CH_2_), 7.50 (s, 1H, Imd CH).

^13^C NMR (100.6 MHz, CDCl_3_, 25 °C): δ, ppm: 162.35, 159.97, 139.42, 136.87, 125.11, 72.59, 72.00, 70.69, 70.66, 68.98, 68.77, 64.77, 64.34, 61.82, 59.15, 47.53, 32.02, 31.09, 29.78, 29.75, 29.71, 29.64, 29.55, 29.46, 29.17, 26.58, 22.80, 14.25.

IR (KBr) ν_max_ cm^−1^: 2925 (C-H alk), 1719 (C=O), 1538 (Imd), 1456 (CH**_3_** alk), 1218 (C(O)-O), 1107 (C-O-C).

HR ESI MS (m/z) [M+H]^1+^ calcd. for C_33_H_61_N_2_O_10_^1+^ = 645.4321, found 645.4320, [M+Na]^1+^ calcd. for C_33_H_60_N_2_NaO_10_^1+^ = 667.4140, found 667.4121.

Synthesis of 4,5-bis[2-(2-(2-methoxyethoxy)ethoxy)ethoxycarbonyl]-1,3-dimethyl-imidazolium iodide (**3a**) and 4,5-bis[2-(2-(2-methoxyethoxy)ethoxy)ethoxycarbonyl]-1-methyl-3-tetradecylimidazolium iodide (**3b**).

A GlassChem reactor was charged with acetonitrile (3 mL), and then 4,5-bis[(2-(2-(2-methoxy) ethoxy)ethoxy)ethoxycarbonyl]-1-methylimidazole (0.8 g, 0.4 mmol) (or 4,5-bis[(2-(2-(2-methoxy)ethoxy)ethoxy)ethoxycarbonyl]-1-tetradecylimidazole (0.17 g, 0.27 mmol) for **3b**) and CH_3_I (0.14 mL, 2.2 mmol for **3a** or 0.08 mL, 1.3 mmol for **3b**) were added. The mixture was flushed with nitrogen and held under stirring at 60 °C for 29 h (for 18 h in the case of **3b**).

For **3a,** the reaction mixture was evaporated under reduced pressure, and the residue was extracted from water (5 mL) with hexane (4 × 7 mL) and then with Et_2_O (3 × 7 mL). The water layer was evaporated, and the product was obtained as a yellow oil. The yield was 0.21 g (80%) and the TLC R_f_ = 0.09 (CHCl_3_: MeOH, 10:1).

For **3b,** the reaction mixture was evaporated, and the product was obtained as a yellow oil. The yield was 0.2 g (95%) and the TLC R_f_ = 0.19 (CHCl_3_: MeOH, 10:1).

**3a**^1^H NMR (400 MHz, CDCl_3_, 25 °C): δ, ppm: 3.37 (s, 6H, OCH_3_), 3.51–3.55 (m, 4H, CH**_3_**-O-CH_2_), 3.59–3.68 (m, 12H, OCH_2_), 3.78 (brt, J = 4.5, 4H, COOCH_2_CH_2_), 4.20 (s, 3H, N-CH_3_), 4.57 (brt, J = 4.4 Hz, 4H, COOCH_2_), 11.09 (s, 1H, Imd CH).

^13^C NMR (100.6 MHz, CDCl_3_, 25 °C): δ, ppm: 156.39, 141.08, 127.30, 71.77, 70.44, 70.40, 68.21, 66.20, 59.00.

IR (KBr) ν_max_ cm^−1^: 2885 (C-H), 1736 (C=O), 1568 (Imd), 1452 (CH_3_) 1258 (C(O)-O), 1108 (C-O-C).

HR ESI MS (m/z) [M-I]^1+^ calcd. for C_21_H_37_N_2_O_10_^1+^ = 447.2448, found 447.2446.

**3b** ^1^H NMR (400 MHz, CDCl_3_, 25 °C): δ, ppm: 0.87 (t, 3H, J = 6.6 Hz, C_14_ CH_3_), 1.21–1.41 (m, 22H, C_14_ CH_2_), 1.89–1.98 (m, 2H, N-CH_2_-CH_2_), 3.38 (s, 6H, O-CH_3_), 3.52–3.55 (m, 4H, CH_3_-O-CH_2_), 3.60–3.68 (m, 12H, O-CH_2_), 3.76–3.80 (m, 4H, C(O)-O-CH_2-_CH_2_), 4.23 (s, 3H, N-CH_3_), 4.49–4.58 (m, 6H, N-CH_2_, C(O)-O-CH_2_), 11.12 (s, 1H, Imd CH).

^13^C NMR (100.6 MHz, CDCl_3_, 25 °C): δ, ppm: 156.54, 156.37, 140.50, 127.19, 126.93, 71.85, 70.51, 68.23, 66.44, 66.27, 59.04, 50.29, 37.29, 31.88, 30.36, 29.62, 29.52, 29.40, 29.33, 28.95, 26.25, 22.65, 14.11.

IR (KBr) ν_max_ cm^−1^: 2923 (C-H alk), 1737 (C=O), 1562 (Imd), 1467 (CH_3_ alk), 1253 (C(O)-O), 1107 (C-O-C).

HR ESI MS (m/z) [M-I]^1+^ calcd for C_34_H_63_N_2_O_10_^1+^ = 659.4478, found: 659.4478; [M-I-C_6_H_13_O_3_]^1+^ calcd for C_28_H_51_N_2_O_7_^1+^ = 527.3691, found: 527.3680

Synthesis of trans-{4,5-bis[2-(2-(2-methoxyethoxy)ethoxy)ethoxycarbonyl]-1,3-dimethylimidazolin-2-ylidene}{pyridine}palladium(II) diiodide (**4a**) and trans-{4,5-bis[2-(2-(2-methoxyethoxy)ethoxy)ethoxycarbonyl]-1-methyl-3-tetradecylimidazolin-2-ylidene}{pyridine}palladium(II) diiodide (**4b**).

A GlassChem reactor was charged with 4,5-bis[2-(2-(2-methoxyethoxy)ethoxy)ethoxycarbonyl]-1,3-dimethylimidazolium iodide (0.1 g, 0.17 mmol for **4a**) or 4,5-bis[2-(2-(2-methoxyethoxy)ethoxy)ethoxycarbonyl]-1-methyl-3-tetradecylimidazolium iodide (0.15 g, 0.19 mmol for **4b**), KI (0.11 g, 0.66 mmol for **4a** or 0.13 g, 0.76 mmol for **4b**), and K_2_CO_3_ (0.11 g, 0.83 mmol for **4a** or 0.13 g, 0.95 mmol for **4b**). Pyridine (2 mL for **4a** or 2.5 mL for **4b**) was added followed by the addition of Pd(OAc)_2_ (0.037 g, 0.17 mmol for **4a** or 0.043 g, 0.19 mmol for **4b**). The reaction mixture was flushed with nitrogen and held under vigorous stirring at room temperature for 30 min and then at 65 °C for 1 h. After cooling to room temperature, the reaction mixture was diluted with CH_2_Cl_2_, and the inorganic precipitate was filtered.

For **4a,** the filtrate was evaporated under reduced pressure, and the residue was purified by column chromatography using chloroform–acetone (20:1) as the eluent. After evaporating, the eluent product was obtained as an orange oil. The yield was 0.08 g (50%) and the TLC R_f_ = 0.57 (CHCl_3_: MeOH, 10:1).

For **4b,** the filtrate was evaporated under reduced pressure, and the residue was purified by column chromatography using ethyl acetate as the eluent. After evaporating, the eluent product was obtained as an orange oil. The yield was 0.14 g (67%) and the TLC R_f_ = 0.69 (CHCl_3_: MeOH, 10:1).

**4a**^1^H NMR (400 MHz, CDCl_3_, 25^o^C): δ, ppm: 3.38 (s, 6H, OCH_3_), 3.53–3.57 (m, 4H, CH_3_-O-CH_2_), 3.61–3.68 (m, 12 H, OCH_2_), 3.76(brt, J = 4.8 Hz, 4H, COOCH_2_-CH_2_), 4.16 (s, 3H, N-CH_3_), 4.47 (brt, J = 4.4 Hz, 4H, COOCH_2_), 7.34 (t, 2H, J = 6.8, Py N-CH-CH), 7.76 (t, 1H, J = 7.6 Hz, Py N-CH-CH-CH), 9.02 (d, 2H, J = 5.0 Hz, Py N-CH).

^13^C NMR (100.6 MHz, CDCl_3_, 25 °C): δ, ppm: 158.13, 156.03, 153.97, 137.94, 128.76, 124.70, 72.01, 70.67, 70.39, 68.62, 65.46, 59.18.

IR (KBr) ν_max_ cm^−1^: 2880 (C-H), 1726 (C=O), 1602 (Py), 1446 (CH**_3_**) 1252 (C(O)-O), 1102 (C-O-C), 1017 (Py).

HR ESI MS (m/z) [M-I-C_5_H_5_N]^1+^ calcd for C_21_H_36_IN_2_O_10_Pd^1+^ = 709.0444, found: 709.0445, [M-C_5_H_5_N+NH_4_]^1+^ calcd for C_21_H_40_I_2_N_3_O_10_Pd^1+^ = 853.9832, found: 853.9829, [M-C_5_H_5_N+NH_4_+CH_3_CN]^1+^ calcd for C_23_H_43_I_2_N_4_O_10_Pd^1+^ = 895.0098, found: 895.0102.

**4b** ^1^H NMR (400 MHz, CDCl_3_, 25^o^C): δ, ppm: 0.87 (t, J = 6.4 Hz, 3H, CH_2_-CH_3_), 1.21–1.35 (m, 20H, CH_2_ C_14_), 1.43 (brs, 2H, N-CH_2_-CH_2_-CH_2_), 2.06–2.15 (m, 2H, N-CH_2_-CH_2_), 3.38 (s, 6H, O-CH_3_), 3.53–3.57 (m, 4H, CH_3-_O-CH_2_), 3.61–3.68 (m, 12H, O-CH_2_), 3.73–3.78 (m, 4H, COOCH_2-_CH_2_), 4.17 (s, 3H, N-CH_3_), 4.43–4.49 (m, 4H, COOCH_2_), 4.65 (brt, J = 7.9 Hz, 2H, N-CH_2_), 7.34 (t, J = 6.5 Hz, 2H, N-CH-CH Py), 7.76 (t, J = 7.6 Hz, 1H, N-CH-CH-CH Py), 9.01 (d, 2H, J = 5.1 Hz, N-CH Py).

^13^C NMR (100.6 MHz, CDCl_3_, 25 °C): δ, ppm: 158.45, 158.35, 155.30, 154.00, 137.89, 128.77, 128.49, 124.68, 72.02, 70.68, 65.50, 65.40, 59.18, 51.56, 39.23, 32.03, 29.81, 29.47, 29.32, 29.24, 26.93, 22.80, 14.24.

IR (KBr) ν_max_ cm^−1^: 2923(C-H alk), 1727 (C=O), 1588 (Py), 1447 (CH_3_ alk), 1249 (C(O)-O), 1109 (C-O-C), 1027 (C-H Py).

HR ESI MS (m/z) [M-I-C_5_H_5_N]^1+^ calcd. for C_34_H_62_IN_2_O_10_Pd^1+^ = 891.2484, found 891.2463, [M-C_5_H_5_N+NH_4_]^1+^ calcd. for C_34_H_66_I_2_N_3_O_10_Pd^1+^ = 1036.1866, found 1036.1837, [M-C_5_H_5_N+NH_4_+CH_3_CN]^1+^ calcd. for C_36_H_69_I_2_N_4_O_10_Pd^1+^ = 1077.2132, found 1077.2102.

### 2.3. Microscopy

TEM was performed on a Hitachi HT7700 ExaLens (Hitachi High-Tech Corporation, Tokyo, Japan) device in the Interdisciplinary Center for Analytical Microscopy of Kazan Federal University. The images were acquired at an accelerating voltage of 100 kV. Samples were ultrasonicated in water for 10 min, dispersed on 200-mesh copper grids with continuous formvar support films, and then dried during 3 h in vacuo.

### 2.4. Dynamic Light Scattering

DLS experiments were carried out on a Zetasizer Nano ZS instrument (Malvern Panalytical, Worcestershire, UK) with a 4 mW 633 nm He–Ne laser light source and a light scattering angle of 173°. The data were treated with the DTS software (Dispersion Technology Software 5.00). The solutions were filtered through a 0.8 μM filter before the measurements to remove dust. The experiments were carried out in disposable plastic cells DTS 0012 at 298K with at least three experiments for each system.

### 2.5. Model Reduction Reaction

A total of 5 μL of **5a**,**b**, **4a**,**b**, and **3b** from stock THF solution with C = 4.8 mM and 5 μL of nitroarene from stock THF solution with C = 24 mM were added into a quartz cuvette (1 = 1 cm). A total of 0.3 mL THF and 0.9 mL of water were added. Then, 2.2 mg of NaBH_4_ was added to the resulting mixture. Then, the absorption spectra of the resulting solution were recorded in increments of 120 s for *p*-nitrophenol or 90 s for *p*-ethylnitrobenzene at 25 °C using a Shimadzu UV-2600 spectrophotometer equipped with a Shimadzu TCC-100 thermostat (Shimadzu Corporation, Kyoto, Japan).

### 2.6. Model Suzuki-Miyaura Reaction

In a 2 mL vial equipped with a septum and a stirrer bar, 45 mg of *p*-octyloxybrombenzene and 34 mg of phenylboronic acid were dissolved in DMF or a water–DMF 3:1 mixture (0.8 mL). Then, 0.16 µmol of an appropriate catalyst was added, and the solution was purged with argon through a septum. The reaction was heated on a hot plate with a stirrer at 80 °C, with an aliquot being periodically taken for GCMS analysis.

### 2.7. Gas Chromatography-Mass Spectrometry

Gas chromatography–mass spectrometry was performed on a GCMS-QP2010 Ultra gas chromatography mass spectrometer (Shimadzu, Kyoto, Japan) equipped with an HP-5MS column (the internal diameter was 0.32 mm, and the length was 30 m). The parameters were as follows: Helium 99.995% purity was the carrier gas, the temperature of the injector was 250 °C, the flow rate through the column was 2 mL/min, and the thermostat temperature program was a gradient temperature increase from 70 to 250 °C with a step of 10 °C/min. The range of the scanned masses was *m*/*z* 35–400. The internal standard method using dodecane was used for the quantitative analysis.

## 3. Results

### 3.1. Synthesis

**Scheme 2:**
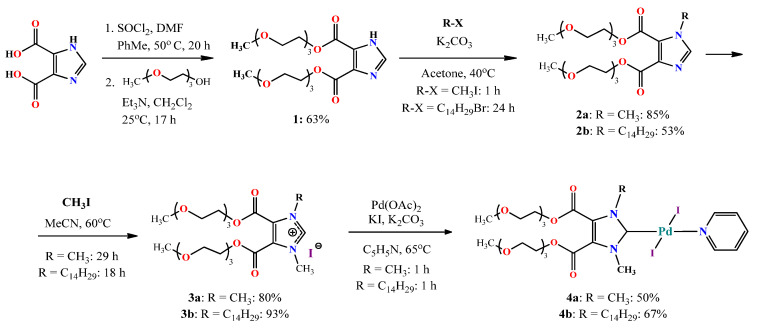
Synthetic pathway for Pd PEPPSI complexes 4 (**a**,**b**).

At the first stage, 1H-imidazole-4,5-dicarboxylic acid, obtained by benzimidazole oxidation [26], was transferred to the acid chloride by treatment with thionyl chloride [27], which was then reacted without additional purification with an excess of thriethylene glycol monomethyl ether (Scheme 2). The resulting product was isolated with a 63% yield. The structure of bis-ester **1** was proven using NMR ^1^H, ^13^C, IR, and high-resolution ESI (HR ESI) mass spectrometry. Due to the equivalence of the two ether fragments in the NMR spectrum, a single set of their signals was observed in the region from 3.3–4.5 ppm. The proton of the imidazolium fragment appeared at 7.85 ppm (Figure S1, a). In the FT IR spectrum, an intense vibrational signal corresponding to the stretching vibrations of the carbonyl group was observed at 1727 cm^−1^. Singly charged cations [M+Na]^1+^, [M+H]^1+^, [M-H+2Na]^1+^, and [M+K]^1+^ were found in the HR ESI mass spectrum (Figure S1d).

The alkylation of the resulting diester 1 was carried out in acetone using methyl iodide or tetradecyl bromide. As expected, the reaction with methyl iodide took significantly less time and the yield was higher (85%). Due to the water solubility, product **2a** was purified by back extraction, and the more-lipophilic **2b** was purified by column chromatography. Due to the nonequivalence of the carbon atoms of the imidazole core as well as ester fragments, in the ^13^C NMR spectrum, five signals in the weak-field region were found for both structures (Figures S2 and S3). The ^1^H NMR spectrum also became more complicated in the region of high fields. In the case of **2a,** the signal of methyl protons appeared as a singlet at 3.84 ppm. In the **2b** ^1^H NMR spectrum, the signals of the tetradecyl fragment protons appeared as triplets at 4.17 and 0.88 ppm as well as a series of multiplets in the region from 1.20–1.33 and 1.71–1.80 ppm. It is noteworthy that, when recording the spectra of **2a**,**b** in CDCl_3_, we found a twofold decrease in the integral intensity of the terminal CH_3_ protons of the glycol fragments. Taking into account the presence of polar glycol groups, we proposed that this was due to the formation of reverse micelle-like aggregates and the following decrease in the relaxation rate of these protons [28]. Indeed, when the spectrum **2a** was conducted again in DMSO-d6, the integral intensity of the CH_3_ protons was found to fully correspond to the structure (Figure S2b). In the HR ESI MS spectrum for both **2a** and b, signals of [M+H]^1+^ and [M+Na]^1+^ were found with m/z = 463.2289 and 485.2105 for **2a** (theoretical m/z = 463.2287 and 485.2106) and m/z = 645.4320 and 667.4121 for **2b** (theoretical m/z = 645.4321 and 667.4140). The resulting alkylated imidazoles **2a** and b were quaternized with methyl iodide in acetonitrile. The resulting quaternized imidazolium salts **3a** and **b** were obtained in high yields. Both the ^1^H NMR and ^13^C spectrums for compound **3a** were significantly simplified due to the symmetry of the latter (Figure S4). For both **3a** and **b,** the proton signal of the imidazolium fragment underwent a significant downfield shift (∆δ = 3.58 for **3a** and 3.70 for **3b**), which was associated with a strong increase in acidity and the loosening of the carbon–hydrogen bond in the imidazolium cycle (Figures S4 and S5). In the HR ESI MS spectrum for both **3a** and **b,** signals of [M-I]^1+^ were found with m/z = 447.2446 and 659.4478 (theoretical m/z = 447.2448 and 659.4478), respectively. The target palladium complexes 4a and b were obtained by heating imidazolium salts **3a**,**b** with palladium acetate, potassium iodide, and potassium carbonate in pyridine for 1 h. Both products were isolated using column chromatography. The ^1^H NMR spectra of both complexes (Figures S6 and S7) showed signals of the pyridine fragment protons with an integrated intensity corresponding to the complex composition. Signals corresponding to the pyridine fragment also appeared in the ^13^C spectrum (153.97, 137.94, and 124.70 ppm for **4a** and 154.00, 137.89, and 124.68 for **4b**), while the signal of the carbene carbon atom underwent a slight downfield shift (∆δ = 15.13 ppm for **4a** and 14.8 ppm for **4b**). The HR ESI MS spectra of both complexes showed a similar pattern of signals. Due to the ease of leaving the pyridine ligand, the signals were recorded with its substitution for ammonia [M-C_5_H_5_N+NH_4_]^1+^and acetonitrile [M-C_5_H_5_N+NH_4_+CH_3_CN]^1+^, which were used as feed phases in the mass spectrometer, with m/z = 853.9829 and 895.0102 for **4a** (theoretical m/z = 895.0098 and 853.9832) and m/z = 1036.1837 and 1077.2102 for **4b** (theoretical m/z = 1036.1866 and 1077.2132). Signals with the elimination of pyridine and one iodine from the palladium coordination sphere [M-I-C_5_H_5_N]^1+^ were also found; m/z = 709.0445 for **4a** (theoretical m/z = 709.0444) and m/z = 891.2463 for **4b** (theoretical m/z = 891.2484). The trans configuration of the resulting complexes **4a**,**b** was suggested from the chemical shifts of the carbene carbon atoms (155–156 ppm), which corresponded to the literature values for trans–NHC complexes with a weakly coordinating pyridine ligand [29,30].

**Figure 1:**
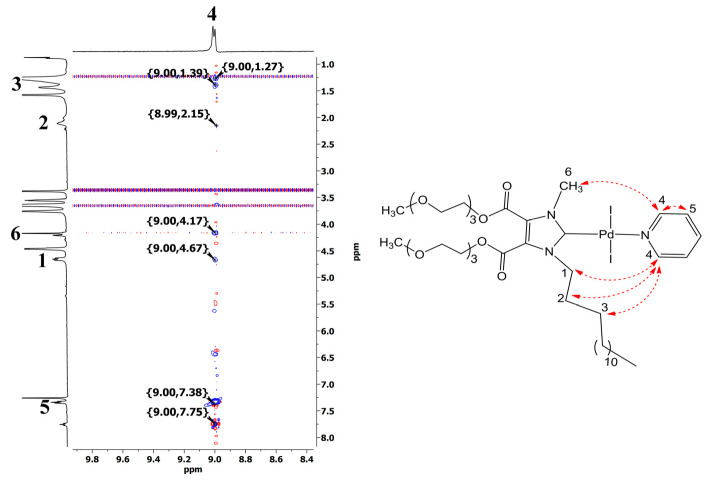
Fragment of 2D NOESY ^1^H-^1^H spectrum of **4b** (CDCl_3_, 25 °C).

The structure of **4b** was also described using 2D NOESY ^1^H-^1^H NMR (Figure 1). Thus, in full agreement with the proposed structure, the spectrum exhibited cross peaks between the proton of the pyridine ring (8.99 ppm) and the protons of the N-methyl group (4.17 ppm) and the methylene protons of the tetradecyl fragment (4.67, 2.15, 1.39, 1.27 ppm).

### 3.2. Catalytic Activities

**Scheme 3:**
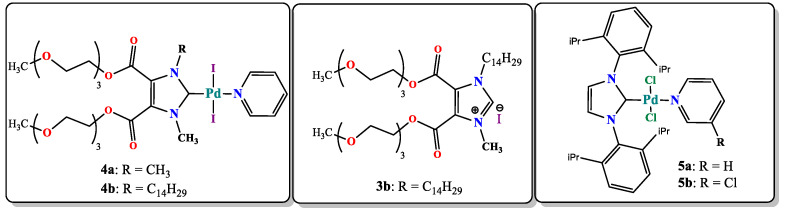
Catalytic systems studied in test reactions.

The next stage of the work was the study of the catalytic activity of the obtained metal complexes. There are two usually used types of NHC-based catalysts according to the type of introduction into the reaction: (1) in situ catalysts, which are made from imidazolium salt and a metal source being directly added to the reaction [31] and (2) pre-formed well-defined complexes, often called precatalysts (“well-defined” precatalysts, which, in the process of catalysis, also form a complex cocktail-type mixture of a metal–NHC complex and NHC, i.e., stabilized metal clusters or nanoparticles [32]). Given the above, we tested both types of systems in a model catalytic reaction. Since Pd–NHC systems are known to be effective catalysts in hydrogenation reactions, including transfer hydrogenation [33,34], complexes **4a**,**b** as well as an in situ catalytic system, obtained from K_2_PdCl_4_ mixed with **3b**, were tested in the model hydrogenation of nitroaromatics. Additionally, for a comparative study, classical Organ’s PEPPSI [35] catalysts containing pyridine or chloropyridine moieties (**5a**,**b**) were also tested (Scheme 3).

**Figure 2:**
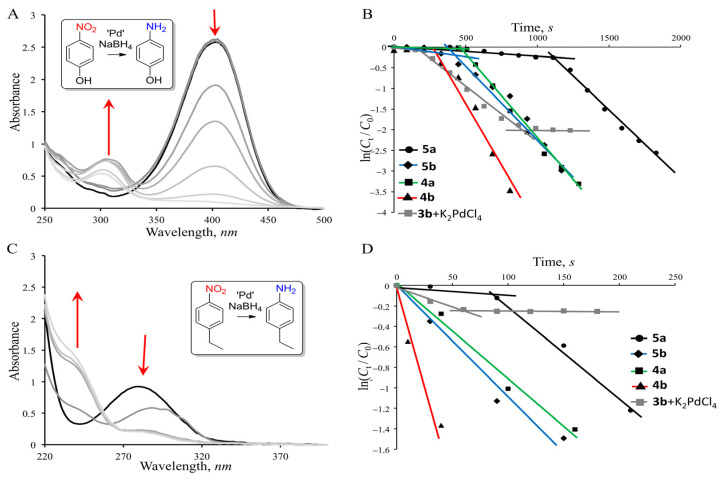
Changes in the UV–VIS spectrum during the reduction of (**A**) *p*-nitrophenol and (**C**) *p*-ethylnitrobenzene in the presence of **4b**; dependence of ln(C_t_/C_0_) vs. t in the presence of different Pd sources: (**B**) for *p*-nitrophenol and (**D**) *p*-ethylnitrobenzene. C (*p*-nitrophenol) = C (*p*-ethylnitrobenzene) = 0.1 mM, C (NaBH_4_) = 5 mM, C (**5a**,**b**; **4a**,**b**, **3b** and K_2_PdCl_4_) = 0.02 mM, H_2_O:THF=3:1, 20 °C, and V = 1.2 mL.

The model catalytic reaction of *p*-nitrophenol reduction in water has proven itself well for assessing relative catalytic activity since it can be easily monitored by changing the absorption band of the chromophore nitro group [36]. Taking into account the amphiphilic nature of the obtained complexes, the more-hydrophobic *p*-ethylnitrobenzene was also used to reveal the micellar effect in the reduction reaction. The reaction proceeded with an excess of NaBH_4_ in a water–THF 3:1 mixture at 20°C in the presence of a 0.02 mM (10 mol% to *p*-nitroarene) catalyst. As examples, Figure 2A,C shows the changes in the UV spectra of a mixture of sodium borohydride and *p*-nitrophenol/*p*-ethylnitrobenzene after the addition of complex **4b**. The absorption band of *p*-nitrophenol at 400 nm decreased, and an absorption band of *p*-aminophenol appeared at 300 nm. For *p*-ethylnitrobenzene, the absorption B-aromatic band at 280 nm decreased, which was accompanied by an increase in the K-aromatic band of *p*-ethylaniline at 234 nm.

Due to the use of a 50-fold excess of NaBH_4_, the reduction process was a pseudo-first-order reaction and is described by the equation −***k_t_*** = ln(***C_t_***/***C*_0_**), where ***C*_0_** and ***C_t_*** are the initial concentration of nitroarene and its concentration at time t, respectively. During the reduction of *p*-nitrophenol, the initial induction period of the reaction (this period is usually ascribed to the diffusion time required for *p*-nitrophenol to be adsorbed onto the catalyst’s surface before the reaction starts [36]) was observed for all the studied catalytic systems (Figure 2B). In the reduction of *p*-ethylnitrobenzene, the induction period was only observed for the **5a** catalyst. As for the in situ system of **3b** + K_2_PdCl_4,_ in both cases the reaction stopped (it reached a plateau without the complete reduction of the nitro derivatives). Table 1 shows the rate constants and specific catalytic activities of the studied catalytic systems.

**Figure 3:**
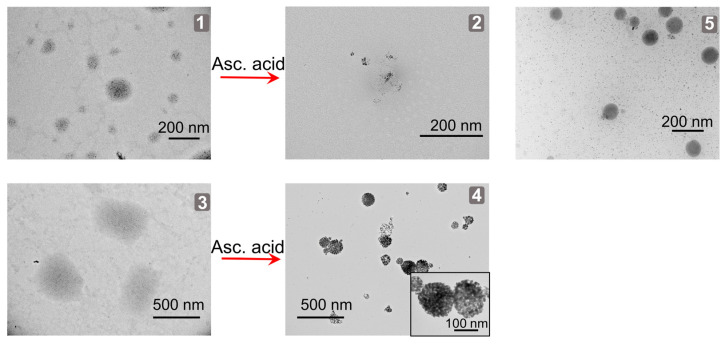
TEM images of **4a** before (1) and after (2) treating with ascorbic acid; **4b** before (3) and after (4) treating with ascorbic acid, and **3b**+K_2_PdCl_4_ (5) treated with ascorbic acid, with different scale bars.

Catalyst **4b** showed the best catalytic efficiency in the reduction reaction with both substrates. The difference between **4b** and the other tested systems was especially noticeable during the reduction of the more-hydrophobic *p*-ethylnitrobenzene. In this case, its rate constant turned out to be three times higher than the other studied systems. The in situ system of **3b** + K_2_PdCl_4_ turned out to be the least effective, which was especially evident in the reduction of *p*-ethylnitrobenzene. Most likely, in the case of the in situ system, after the addition of sodium borohydride, most of the palladium ions were reduced and aggregated into the catalytically inactive palladium black. These results were consistent with the Organ’s catalyst’s previously shown results for Negishi coupling; indeed, the in situ system of PEPPSI complexes was found to be much less efficient compared to the preformed catalysts [35]. To study the difference in the morphology of the system in the presence of a reducing agent, we used transmission microscopy (TEM), studying both the initial catalytic systems and the systems treated with a reducing agent (Figure 3). Ascorbic acid was chosen as a milder reducing agent instead of NaBH_4_, allowing a reduction in the kinetics of the nucleation of palladium particles and the kinetics of their deposition [37] in order to clarify the difference between the various Pd-containing sources (Pd complexes **4a** and **b** and the in situ system of **3b**+K_2_PdCl_4_) in terms of the morphology and distribution of Pd particles.

The images of **4a** and **4b** before treatment with the reducing agent show dotted palladium nanoparticles organized on an organic substrate. The presence of palladium nanoparticles even in the absence of a reducing agent was expected, given the ability of palladium complexes to self-reduce [38]. After the addition of the reducing agent, in both **4a** and **4b**, the formation of palladium clusters was observed, but only in the case of the tetradecyl complex **4b** was their high stabilization and uniform distribution reached. The reduction of palladium ions in the presence of **3a** led to chiseled particles, which were distributed randomly and did not concentrate on the organic support. The data obtained were consistent with the results of the catalysis of the reduction reaction. Using the dynamic light scattering (DLS) method, aqueous solutions of catalysts **4a** and b were studied for the presence of aggregates. The less-lipophilic compound **4a** was found to form aggregates with a polymodal distribution, and the presence of submicron aggregates with 145 nm (45%), 500 nm (38%) and 4450 nm (17%) hydrodynamic diameters and a high polydispersity index of 0.637 (Figure S8) was detected. When the alkyl substituent was elongated, the situation dramatically changed; compound **4b** formed monomodal particles with a hydrodynamic diameter of 194 nm and a polydispersity index of 0.112. Thus, the large difference in the activity of the catalytic system in the reduction reaction, which was especially noticeable in the case of the reaction with *p*-ethylnitrobenzene, was associated with the formation of stable aggregates that could effectively solubilize the hydrophobic substrate, concentrate it, and thereby accelerate the reaction.

Considering that NHC-Pd(II) catalysts are among the best for cross-coupling reactions, we tested the activity of **4ab** and compared it to **5a**,**b** in the Suzuki–Miyaura coupling reaction, which is one of the most convenient model coupling reactions for carrying out in aqueous solutions [39,40], since phenylboronic acid is highly water-soluble. For this, we performed a Suzuki–Miyaura reaction between *p*-octyloxybrombenzene and phenylboronic acid in DMF and in DMF:water 1:3 using a 0.1 mol% Pd catalyst with a gas chromatography–mass spectrometry (GCMS) control using an internal standard (dodecane)**.**

**Figure 4:**
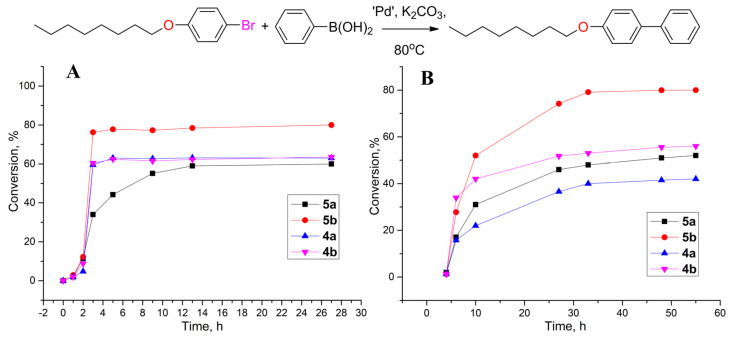
Conversion of *p*-octyloxybrombenzene vs. time in Suzuki–Miyaura reaction between *p*-octyloxybrombenzene and phenylboronic acid in DMF (**A**) or DMF:water 1:3 mixture (**B**). n(*p*-C_8_H_17_OC**_6_**H**_4_**Br) = 0.16 mmol, n(PhB(OH)_2_) = 0.28 mmol, n(K_2_CO_3_) = 0.28 mmol, n(**4a**,**b** and **5a**,**b**) = 0.16 µmol, 80 °C, and V = 0.8 mL.

**Scheme 4:**
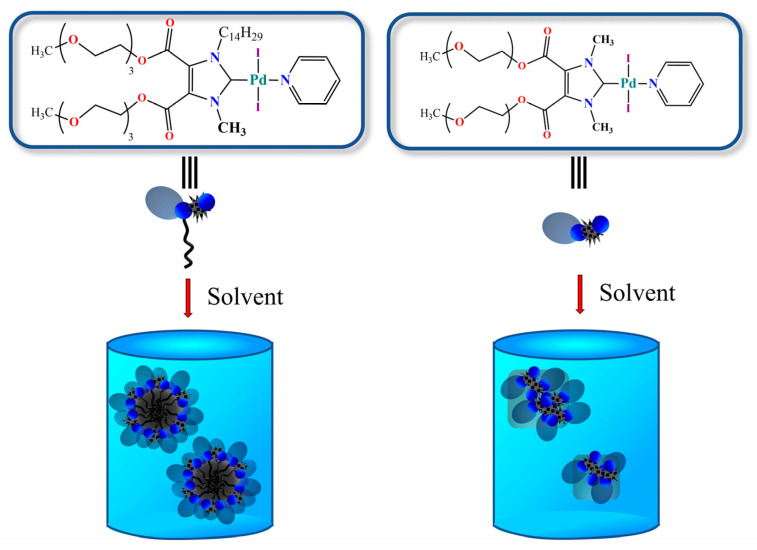
Schematic representation of aggregates formed by systems **4a** and **b** with different lipophilicity.

According to the data obtained (Figure 4), when carrying out the reaction in DMF, for all the tested systems, a selectivity of more than 99% was observed with only trace amounts of biphenyl (a by-product of the homocoupling of phenylboronic acid) being present in the mixture. In the case of all the tested systems, a short induction period was observed in the first 2 h of reaction with a following increase in the reaction rate. The most effective compound in DMF was found to be **5b**, which contained a chloropyridine fragment. Systems **4a**,**b** were found to be similar in DMF, allowing a 60% conversion after 12 h. Catalyst 5a turned out to be the least efficient. Taking into account that the first stage of the catalytic cycle with PEPPSI catalysts is activation, which is achieved due to the dissociation of the pyridine ligand [35], the increased reactivity of the chloropyridine derivative in the reaction was expected, although in the Negishi [41] and sulfination [42] reactions, the pyridine and chloropyridine catalysts showed opposite activities. Upon transition to an aqueous–organic medium, the reaction rate sharply dropped, and the maximum conversion was only achieved after 50 h. There was a big difference between the catalytic systems **4a** and **4b**, which only differed in their lipophilicity. Thus, as in the case of the reduction reaction, the use of the more lipophilic catalyst **4b**, which was capable of forming stable aggregates, led to an increase in the reaction rate (Scheme 4).

## 4. Conclusions

For the first time, a new approach to the synthesis of amphiphilic NHC catalytic systems based on the sequential functionalization of imidazole 4,5-dicarboxylic acid with hydrophilic oligoethylene glycol and lipophilic alkyl fragments was proposed. Complexes of different lipophilicity were obtained, and their catalytic activity was studied in the model reduction and Suzuki–Miyaura reactions. A comparison was made with the most known efficient PEPPSI-type catalytic systems designed by Organ. It was shown that, in the reduction reaction, the most lipophilic complex was three times more active than the commercial PEPPSI complexes, which was associated with the formation of monodisperse aggregates, as detected by the DLS and TEM methods. The resulting system has great prospects for fine tuning both the catalytic activity and the aggregation ability of the system.

## Figures and Tables

**Table 1:** Catalytic activities of different Pd-containing catalytic systems for the reduction of *p*-nitrophenol and *p*-ethylnitrobenzene ^1^.

System	*p*-Nitrophenol		*p*-Ethylnitrobenzene	
	Apparent Rate Constant, *k,* s^−1^	Specific Catalytic Activity, *K_a_*, ×10^5^ mol^1^s^−1^	Apparent Rate Constant, *k,* s^−1^	Specific Catalytic Activity, *K_a_*, ×10^5^ mol^1^s^−1^
**5a**	3.4 × 10^−3^	1.7	12 × 10^−3^	6.0
**5b**	3.5 × 10^−3^	1.75	10 × 10^−3^	5.1
**4a**	4.1 × 10^−3^	2.05	9.2 × 10^−3^	4.6
**4b**	6.7 × 10^−3^	3.35	32 × 10^−3^	16.3
**3b** + K_2_PdCl_4_	2.7 × 10^−3^	1.35	2.8 × 10^−3^	1.4

^1^ C (*p*-nitrophenol) = C (*p*-ethylnitrobenzene) = 0.1 mM, C (NaBH_4_) = 5 mM, C (**5a**,**b**; **4a**,**b**, **3b** and K_2_PdCl_4_) = 0.02 mM, H_2_O:THF=3:1, 20 °C, and V = 1.2 mL.

## Data Availability

Data is contained within the article or supplementary material.

## Supplementary Materials

The following supporting information can be downloaded at: https://www.mdpi.com/article/10.3390/nano12224100/s1, Figure S1: NMR ^1^H (a), ^13^C (b), FT IR (c), and HRESI MS (d) spectra of 4,5-bis-[2-(2-(2-methoxyethoxy)ethoxy)ethoxycarbonyl]imidazole (**1**); Figure S2: NMR ^1^H in CDCl_3_(a), NMR ^1^H in dmso-d6 (b), ^13^C (c), FT IR (d), and HRESI MS (e) spectra of 4,5-bis-[2-(2-(2-methoxyethoxy)ethoxy) ethoxycarbonyl]-1-methylimidazole (**2a**); Figure S3: NMR ^1^H (a), ^13^C (b), FT IR (c), and HRESI MS (d) spectra of 4,5-bis-[2-(2-(2-methoxyethoxy)ethoxy)ethoxycarbonyl]-1-tetradecylimidazole (**2b**); Figure S4: NMR ^1^H (a), ^13^C (b), FT IR (c), and HRESI MS (d) spectra of 4,5-bis[2-(2-(2-methoxyethoxy)ethoxy)ethoxycarbonyl]-1,3-dimethyl-imidazolium iodide (**3a**); Figure S5: NMR ^1^H (a), ^13^C (b), FT IR (c), and HRESI MS (d) spectra of 4,5-bis[2-(2-(2-methoxyethoxy)ethoxy)ethoxycarbonyl]-1-methyl-3-tetradecylimidazolium iodide (**3b**); Figure S6: NMR ^1^H (a), ^13^C (b), FT IR (c), and HRESI MS (d) spectra of *trans*-{4,5-bis[2-(2-(2-methoxyethoxy)ethoxy)ethoxycarbonyl]-1,3-dimethylimidazolin-2-ylidene}{pyridine}palladium(II) diiodide (**4a**); Figure S7: NMR ^1^H (a), ^13^C (b), FT IR (c), and HRESI MS (d) spectra of *trans*-{4,5-bis[2-(2-(2-methoxyethoxy)ethoxy)ethoxycarbonyl]-1-methyl-3-tetradecylimidazolin-2-ylidene}{pyridine}palladium(II) diiodide (**4b**); Figure S8: DLS intensity vs. size graphs for solutions of **4a** (A) and **4b** (B) in water, C [**4a**,**b**]= 0.02 mM.
