# Supplementary material for: Novel PEPPSI-Type NHC Pd(II) Metallosurfactants on the Base of 1H-Imidazole-4,5-dicarboxylic Acid: Synthesis and Catalysis in Water–Organic Media
Source: Nanomaterials (Basel). 2022 Nov 21;12(22):4100. doi: 10.3390/nano12224100 (PMC9694788; doi:10.3390/nano12224100)
Supplement: Supplementary file 1 [file nanomaterials-12-04100-s001.zip › nanomaterials-2031221-supplementary.pdf]

# Novel PEPPSI-Type NHC Pd(II) Metallosurfactants on the Base of 1H-Imidazole-4,5-Dicarboxylic Acid: Synthesis and Catalysis in Water–Organic Media

**Vladimir Burilov [1,*], Dmitriy Radaev [1], Elza Sultanova [1], Diana Mironova [1], Daria Duglav [1], Vladimir Evtugyn [2], Svetlana Solovieva [3] and Igor Antipin [1]**

[1] Kazan Federal University, 18 Kremlevskaya st., 420008 Kazan, Russia
[2] Interdisciplinary Center for Analytical Microscopy, Kazan Federal University, 18 Kremlevskaya st, 420008 Kazan, Russia
[3] Arbuzov Institute of Organic and Physical Chemistry, FRC Kazan Scientific Center of RAS, 8 Arbuzov str., 420008 Kazan, Russia
* Correspondence: ultrav@bk.ru; Tel.: +7-843-2337344

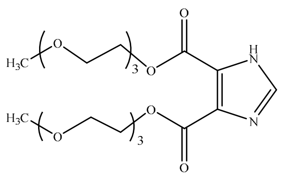
O
H3C
O
3
O
NH
H3C
O
O
3
N
O

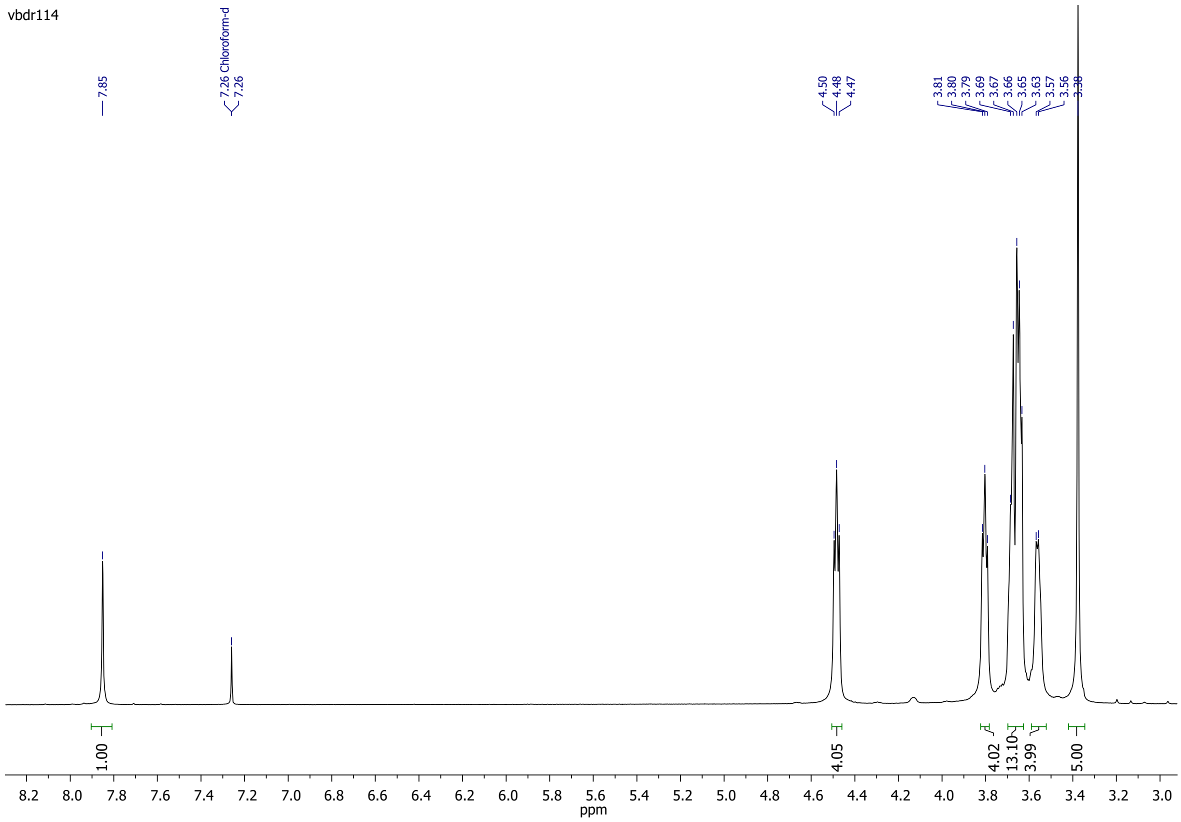
vbdr114
7.85
7.26 Chloroform-d
7.26
4.50
4.48
4.47
3.81
3.80
3.79
3.69
3.67
3.66
3.65
3.63
3.57
3.56
3.38
1.00
4.05
4.02
13.10
3.99
5.00
8.2 8.0 7.8 7.6 7.4 7.2 7.0 6.8 6.6 6.4 6.2 6.0 5.8 5.6 5.4 5.2 5.0 4.8 4.6 4.4 4.2 4.0 3.8 3.6 3.4 3.2 3.0
ppm

**(a)**

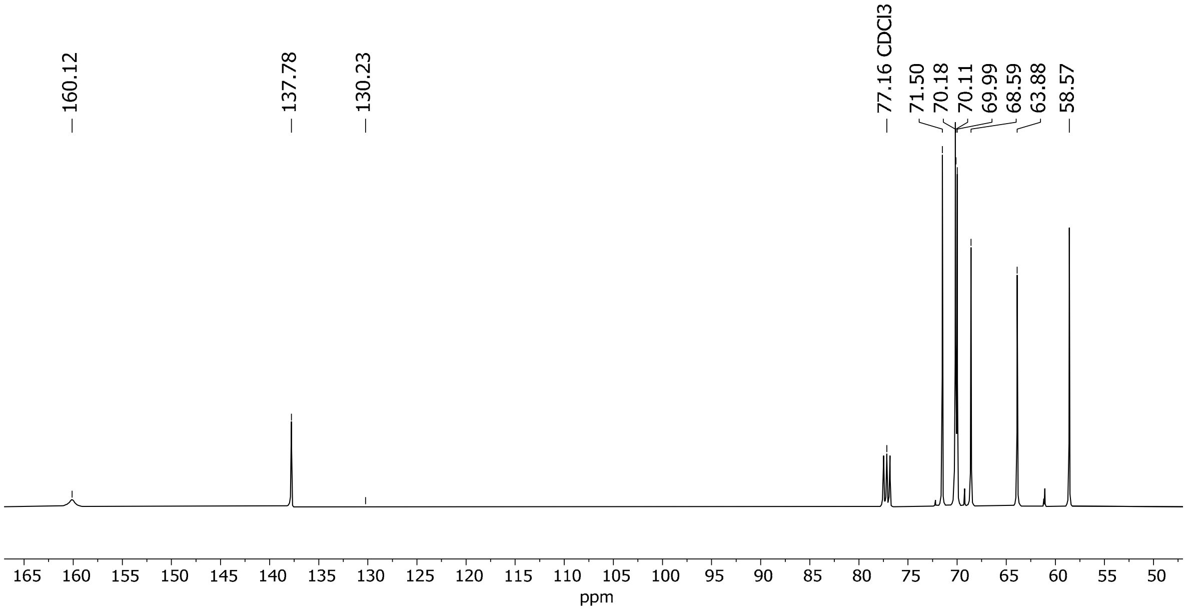
160.12
137.78
130.23
77.16 CDCl3
71.50
70.18
70.11
69.99
68.59
63.88
58.57
165 160 155 150 145 140 135 130 125 120 115 110 105 100 95 90 85 80 75 70 65 60 55 50
ppm

**(b)**

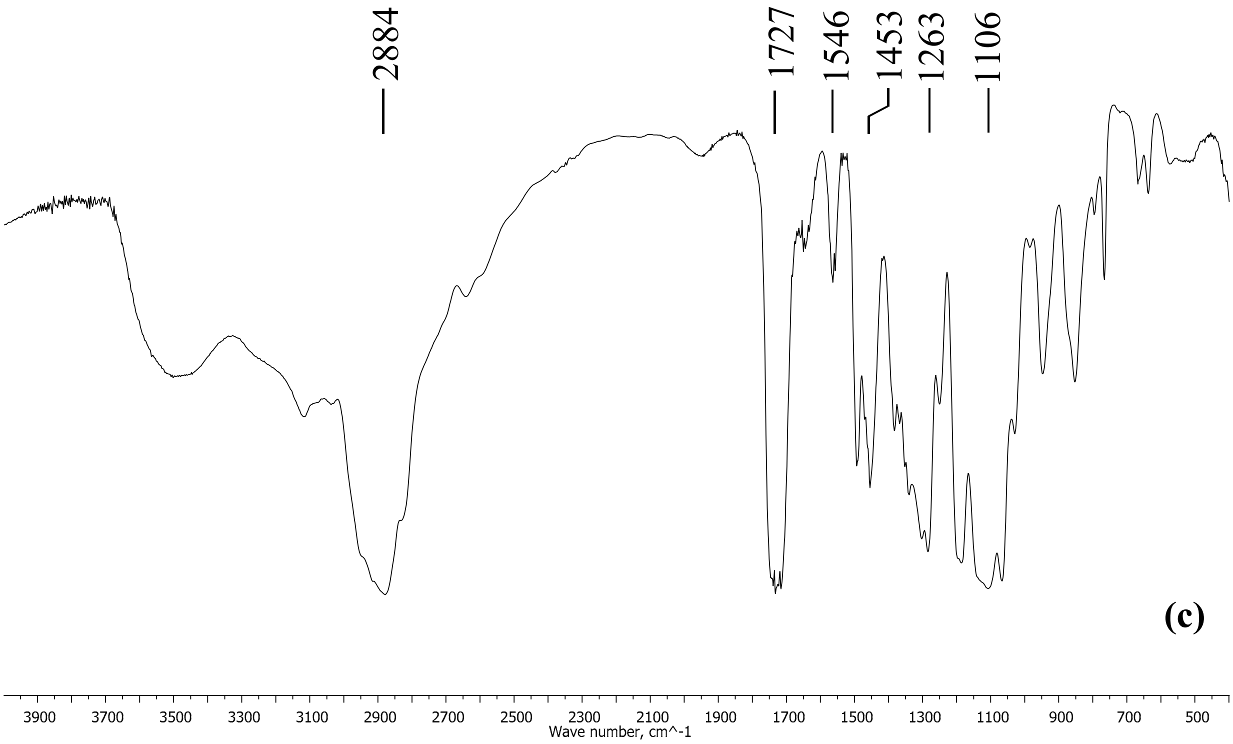


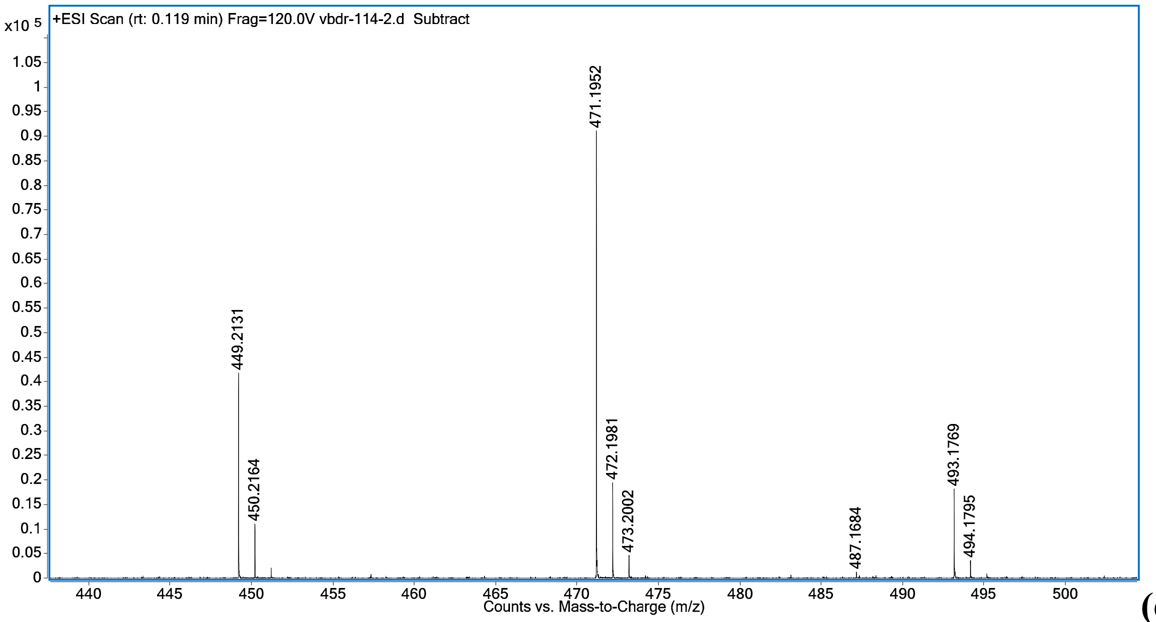


(d)

**Figure S1.** NMR $^{1}$H (a), $^{13}$C (b), FT IR (c) and HRESI MS (d) spectra of 4,5-bis-[2-(2-(2-methoxyethoxy)ethoxy)ethoxycarbonyl]imidazole (**1**).

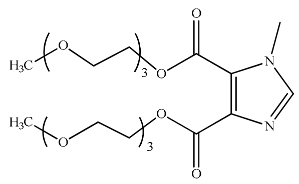

O
O
N
N
O
O
$H_3C$
$H_3C$
3
3


vbdp-24-e

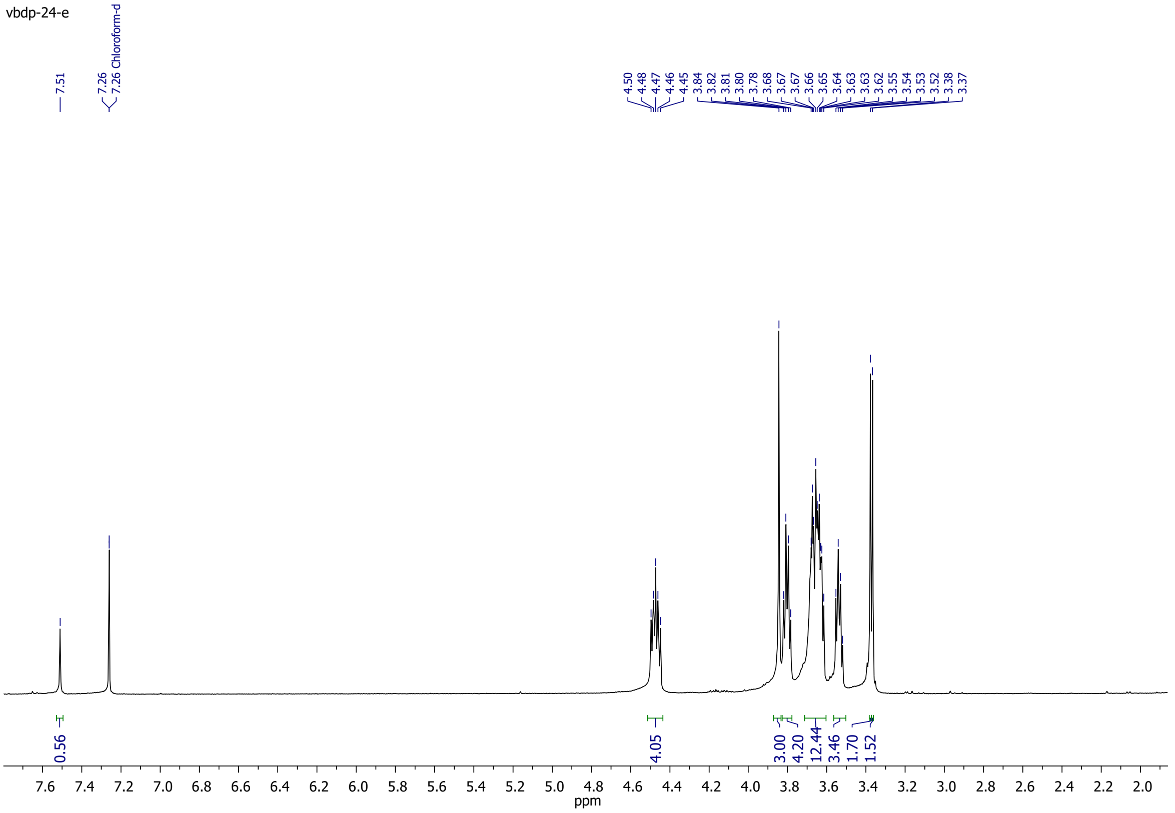

7.51
7.26
7.26 Chloroform-d
4.50
4.48
4.47
4.46
4.45
3.84
3.82
3.81
3.80
3.78
3.68
3.67
3.67
3.66
3.65
3.64
3.63
3.63
3.62
3.55
3.54
3.53
3.52
3.38
3.37
0.56
4.05
3.00
4.20
12.44
3.46
1.70
1.52
7.6 7.4 7.2 7.0 6.8 6.6 6.4 6.2 6.0 5.8 5.6 5.4 5.2 5.0 4.8 4.6 4.4 4.2 4.0 3.8 3.6 3.4 3.2 3.0 2.8 2.6 2.4 2.2 2.0
ppm


**(a)**

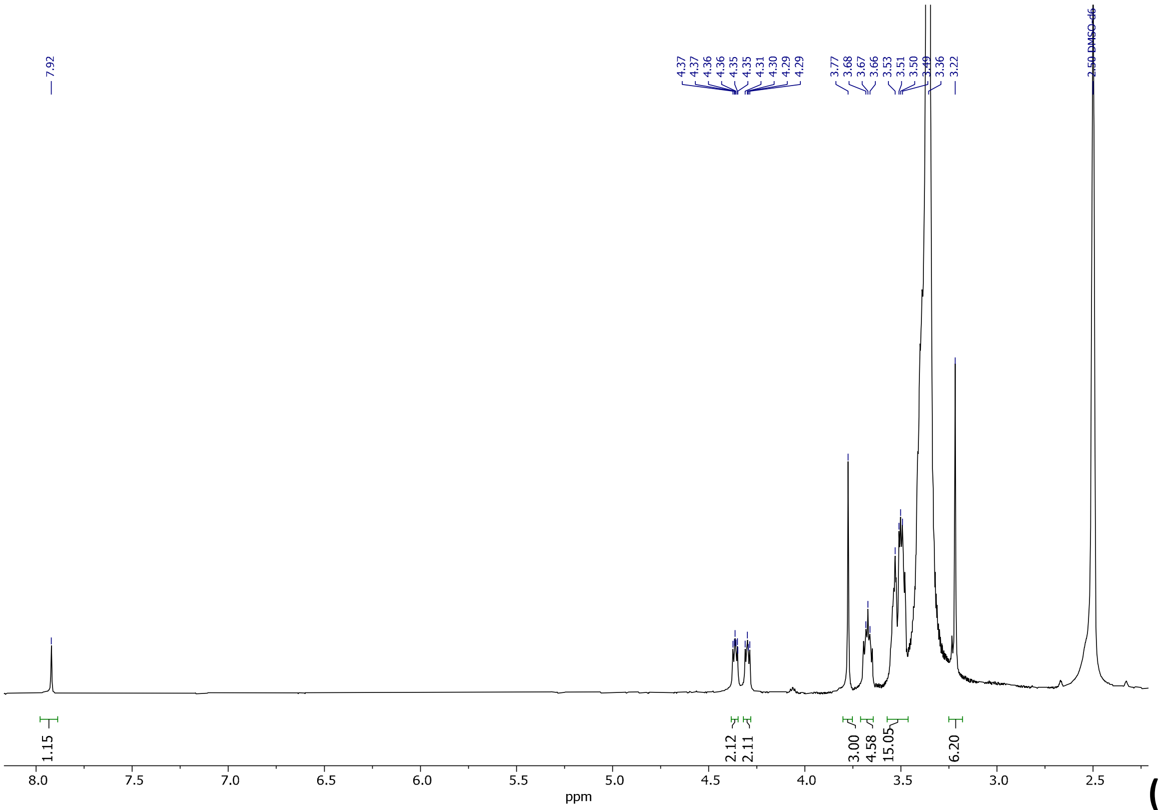

7.92
4.37
4.37
4.36
4.36
4.35
4.35
4.31
4.30
4.29
4.29
3.77
3.68
3.67
3.66
3.53
3.51
3.50
3.49
3.36
3.22
2.50 DMSO-d6
1.15
2.12
2.11
3.00
4.58
15.05
6.20
8.0 7.5 7.0 6.5 6.0 5.5 5.0 4.5 4.0 3.5 3.0 2.5
ppm


**(b)**

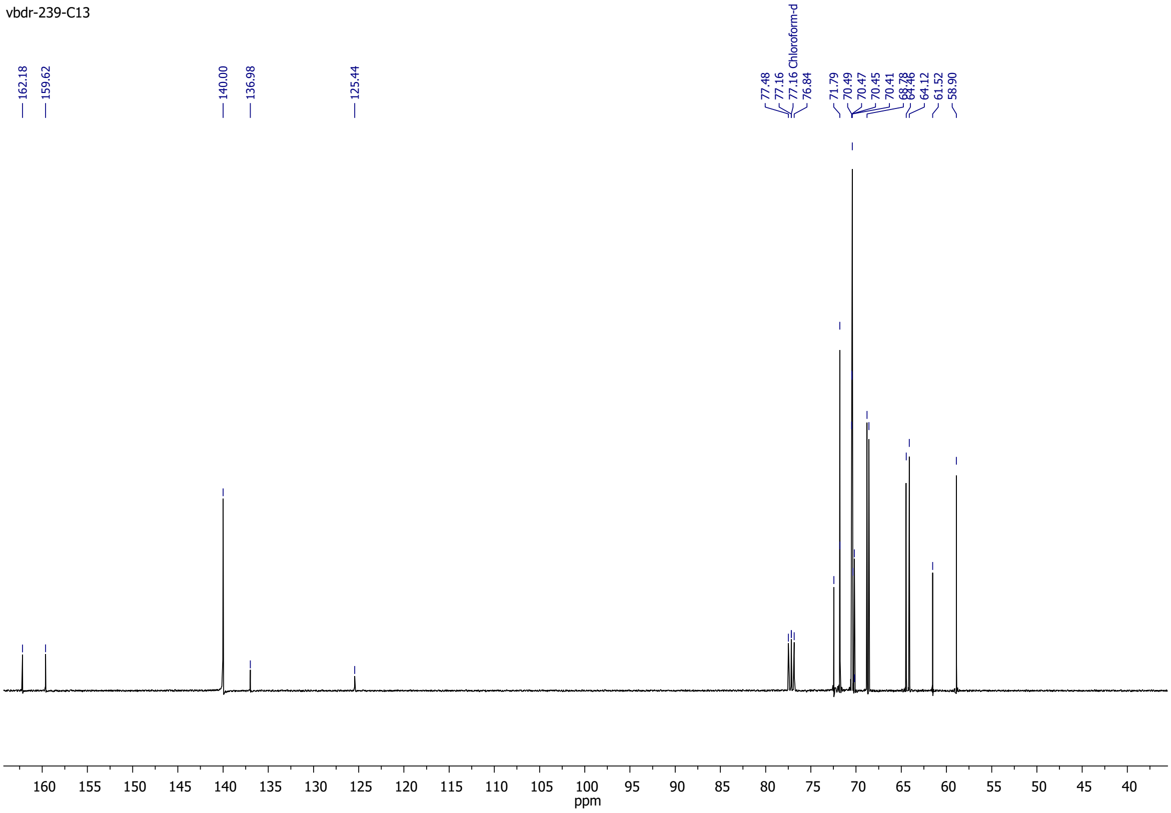
vbdr-239-C13
162.18
159.62
140.00
136.98
125.44
77.48
77.16
77.16 Chloroform-d
76.84
71.79
70.49
70.47
70.45
70.41
64.12
61.52
58.90
ppm

**(c)**

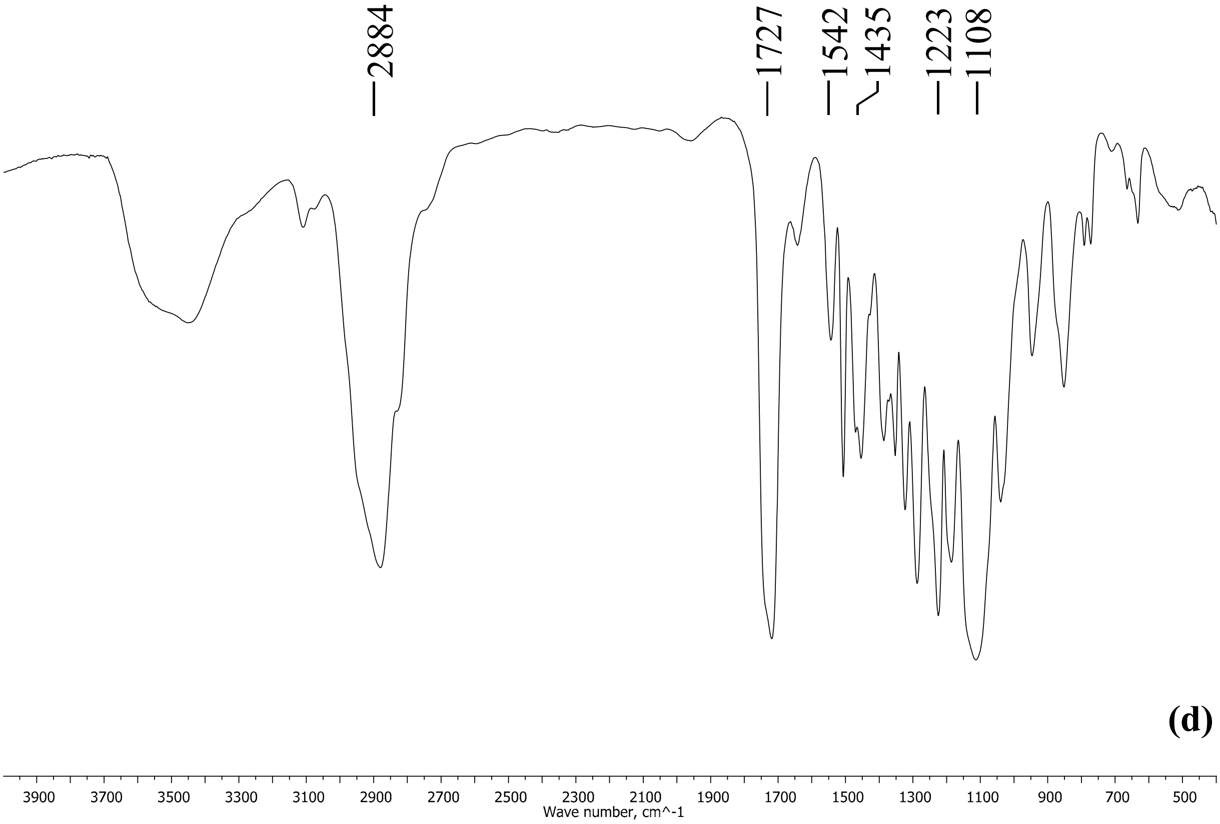
2884
1727
1542
1435
1223
1108
Wave number, cm^-1

**(d)**

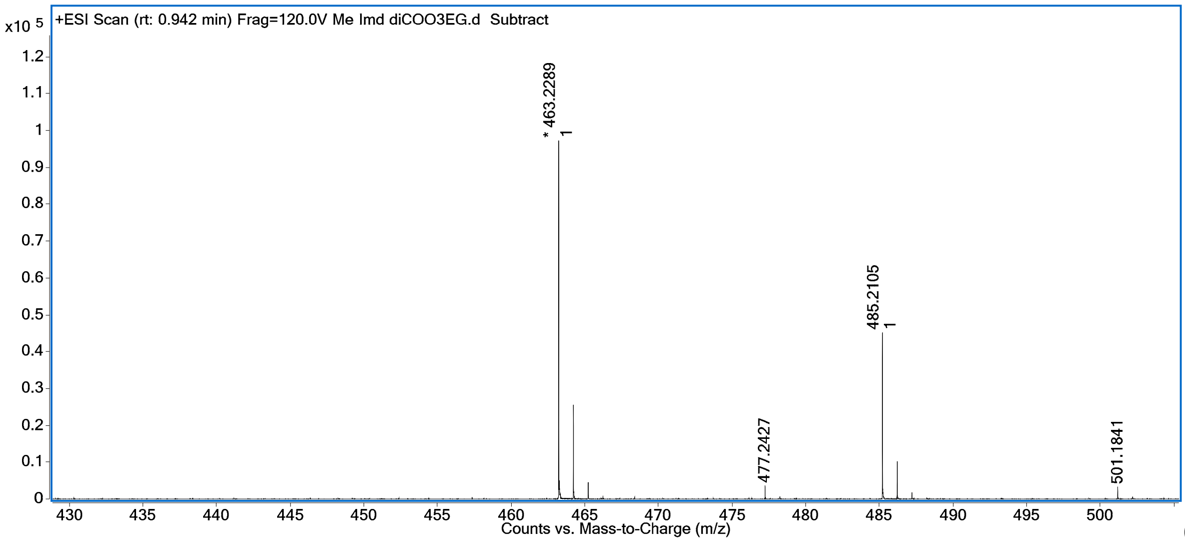


**(d)**

**Figure S2.** NMR $^{1}H$ in $CDCl_3$(a), NMR $^{1}H$ in DMSO-d6 (b), $^{13}C$ (c), FT IR (d) and HRESI MS (e) spectra of 4,5-bis-[2-(2-(2-methoxyethoxy)ethoxy) ethoxycarbonyl]-1-methylimidazole (**2a**)

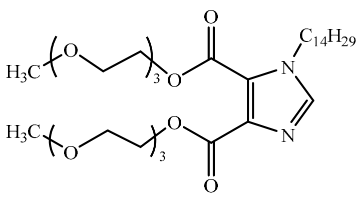
O
C14H29
H3C
O
3
O
N
H3C
O
N
O
3
O

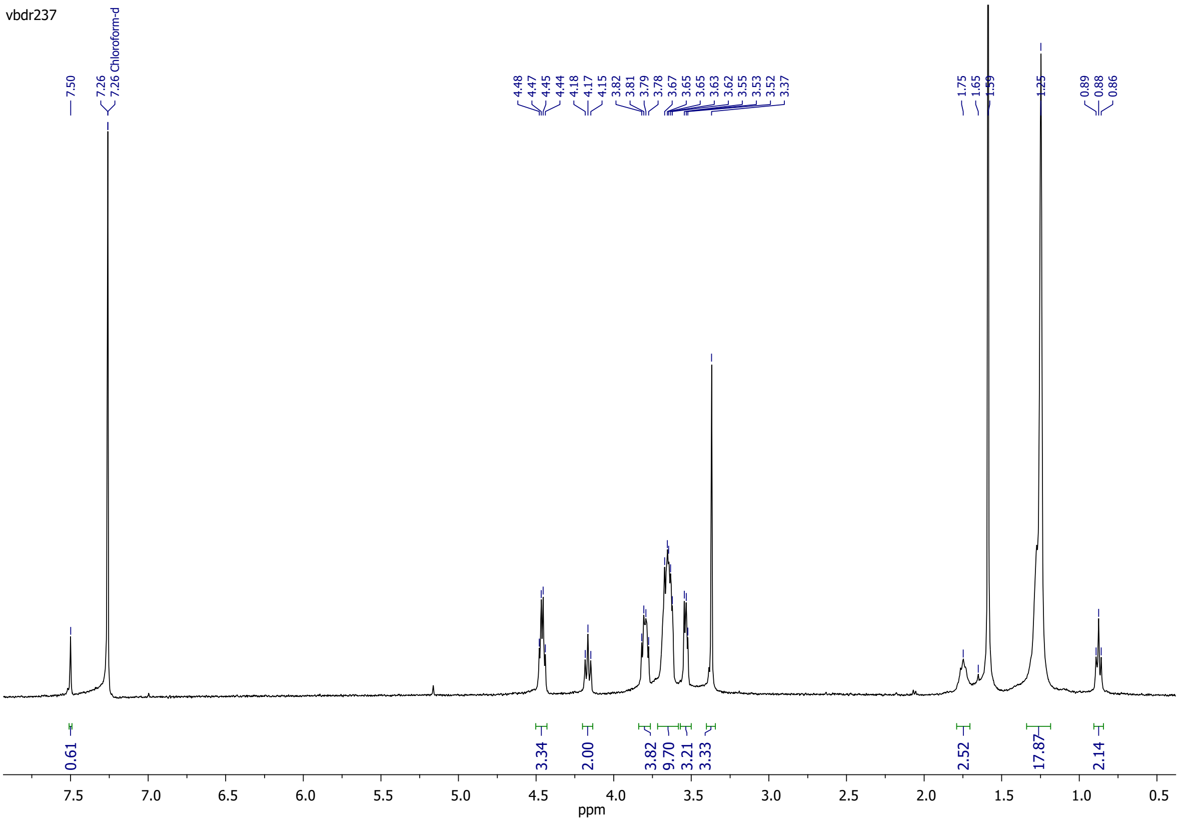
vbdr237
7.50
7.26
7.26 Chloroform-d
4.48
4.47
4.45
4.44
4.18
4.17
4.15
3.82
3.81
3.79
3.78
3.67
3.65
3.65
3.63
3.62
3.55
3.53
3.52
3.37
1.75
1.65
1.59
1.25
0.89
0.88
0.86
0.61
3.34
2.00
3.82
9.70
3.21
3.33
2.52
17.87
2.14
7.5 7.0 6.5 6.0 5.5 5.0 4.5 4.0 3.5 3.0 2.5 2.0 1.5 1.0 0.5
ppm

(a)

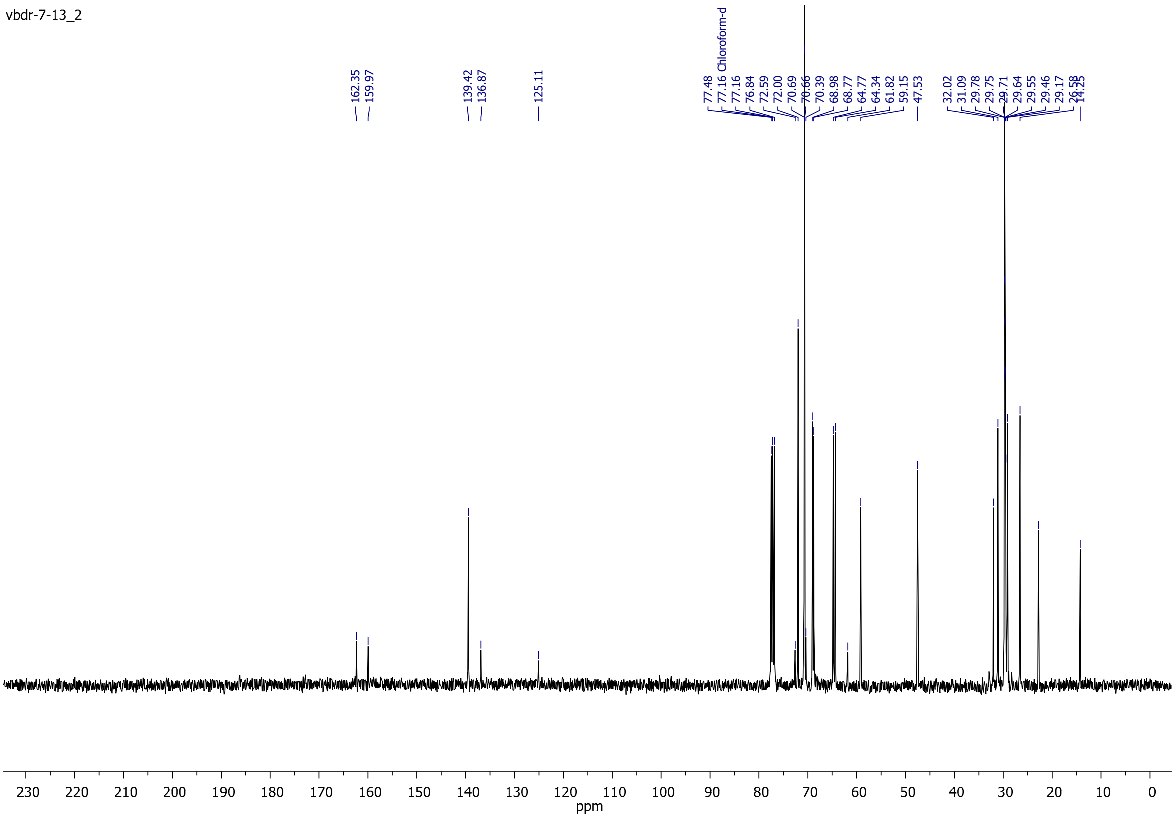
vbdr-7-13_2
162.35
159.97
139.42
136.87
125.11
77.48
77.16 Chloroform-d
77.16
76.84
72.59
72.00
70.69
70.66
70.39
68.98
68.77
64.77
64.34
61.82
59.15
47.53
32.02
31.09
29.78
29.75
29.71
29.64
29.55
29.46
29.17
26.58
14.25
230 220 210 200 190 180 170 160 150 140 130 120 110 100 90 80 70 60 50 40 30 20 10 0
ppm

(b)

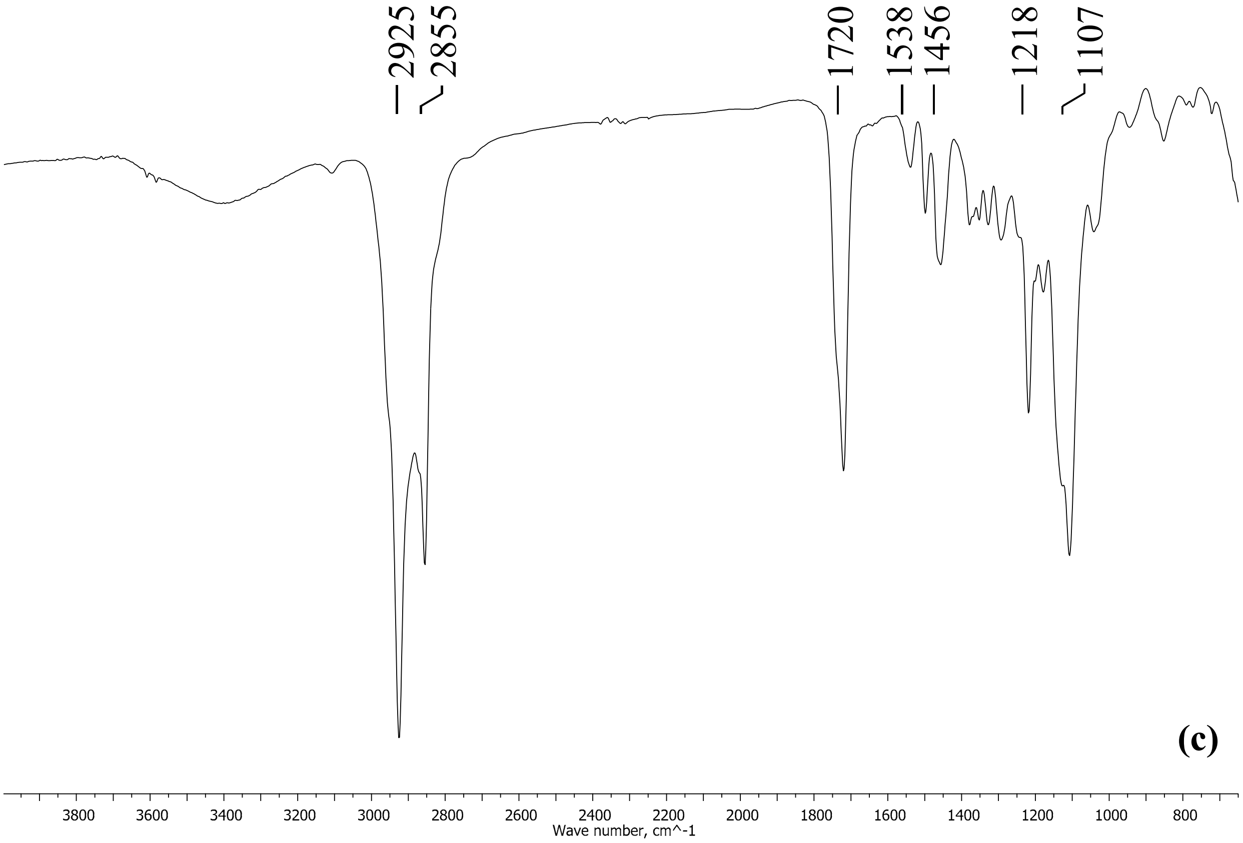


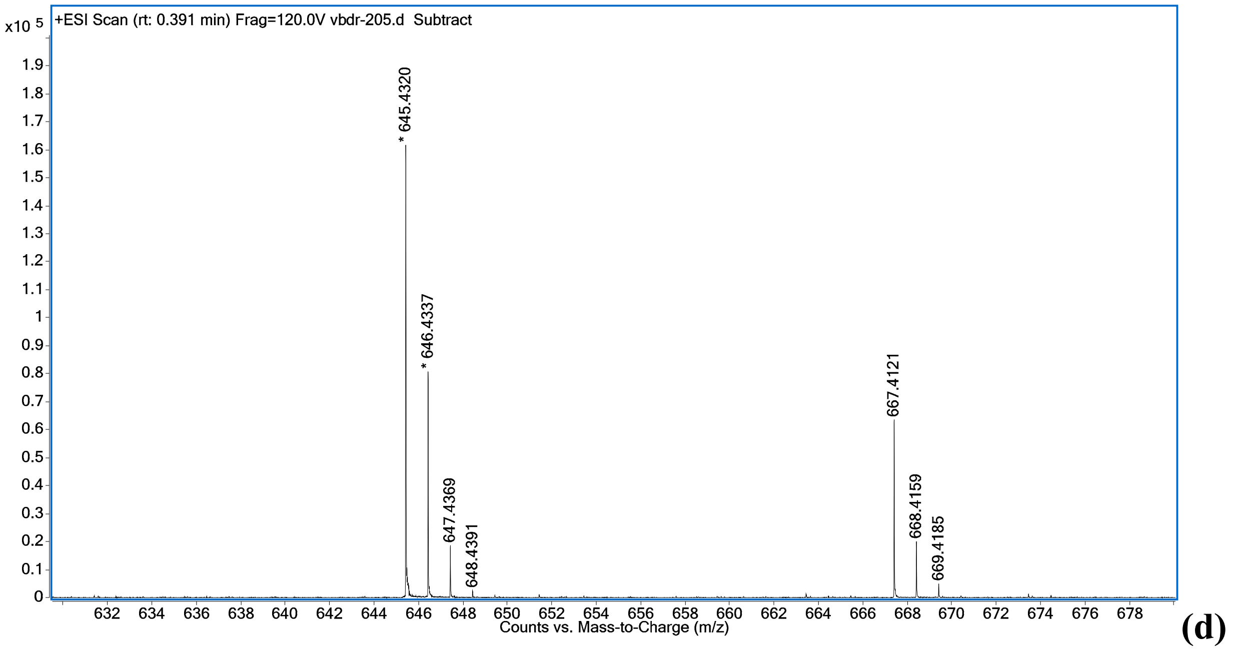


**Figure S3.** NMR $^{1}$H (a), $^{13}$C (b), FT IR (c) and HRESI MS (d) spectra of 4,5-bis-[2-(2-(2-methoxyethoxy)ethoxy)ethoxycarbonyl]-1-tetradecylimidazole (**2b**)

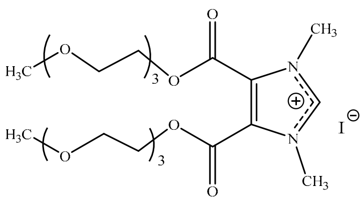
CH3
H3C
O
N
3
I
CH3

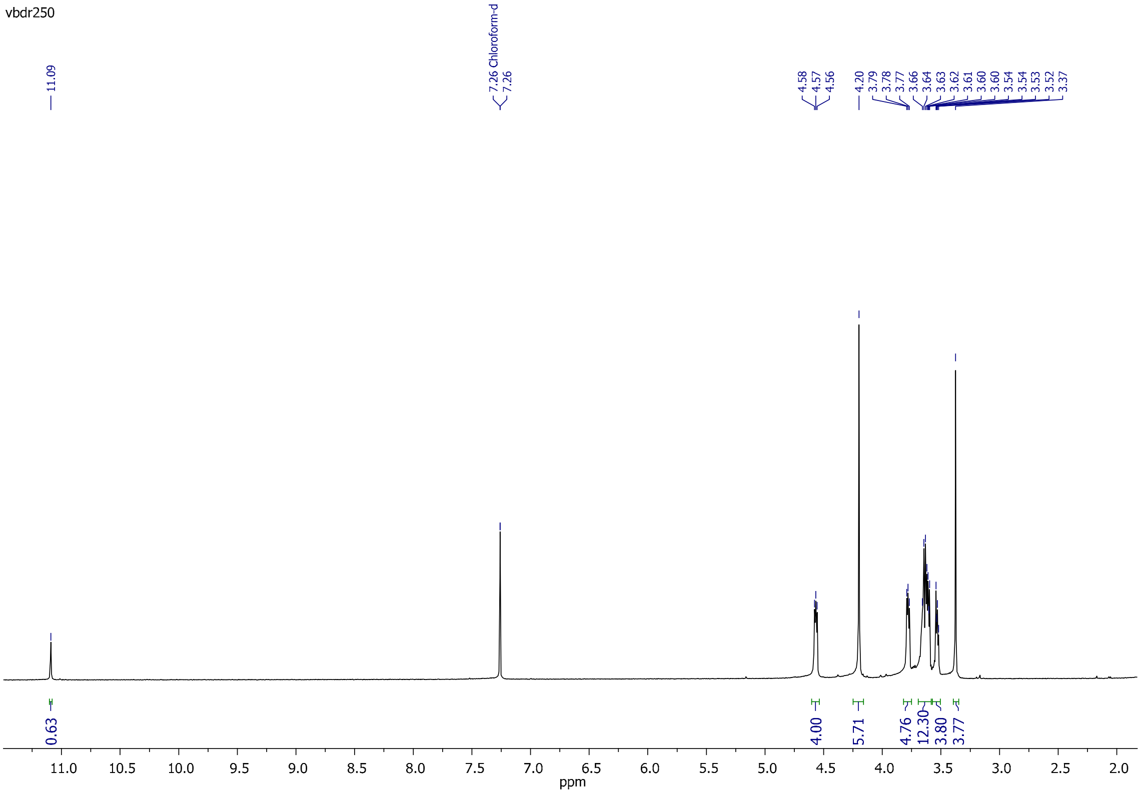
vbdr250
11.09
7.26 Chloroform-d
7.26
4.58
4.57
4.56
4.20
3.79
3.78
3.77
3.66
3.64
3.63
3.62
3.61
3.60
3.60
3.54
3.54
3.53
3.52
3.37
0.63
4.00
5.71
4.76
12.30
3.80
3.77
ppm

(a)

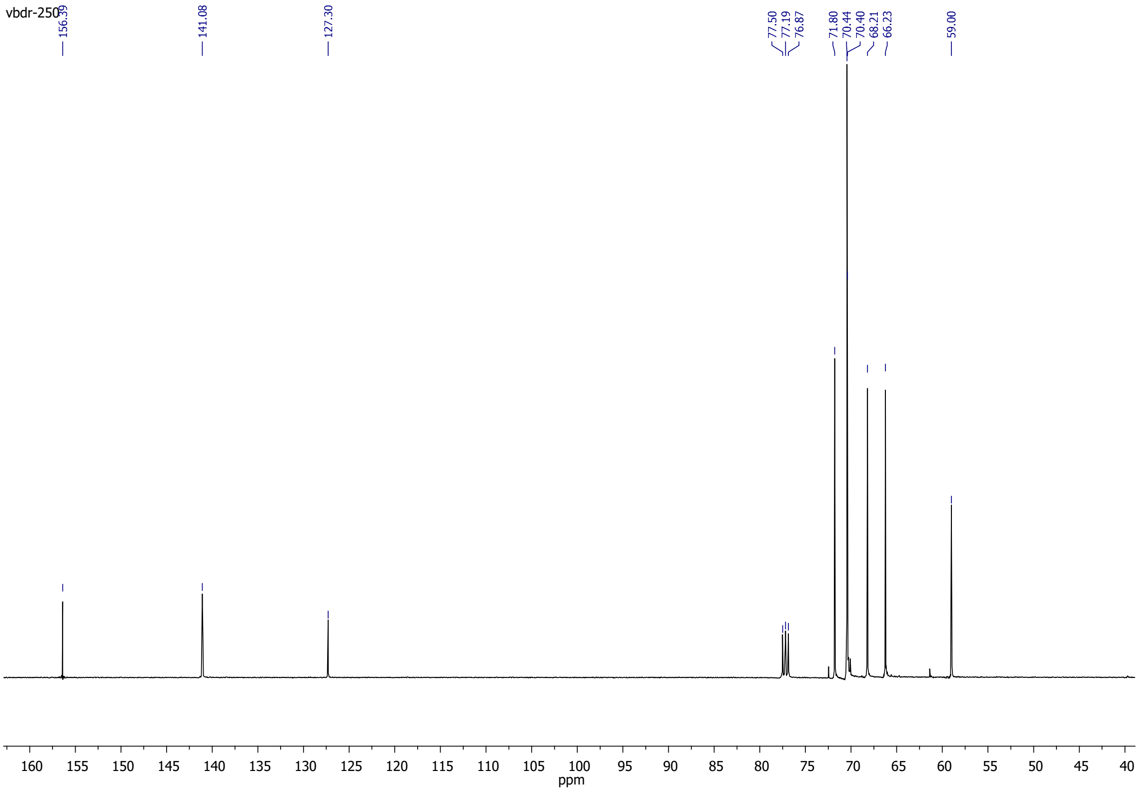
vbdr-250
156.39
141.08
127.30
77.50
77.19
76.87
71.80
70.44
70.40
68.21
66.23
59.00
ppm

(b)

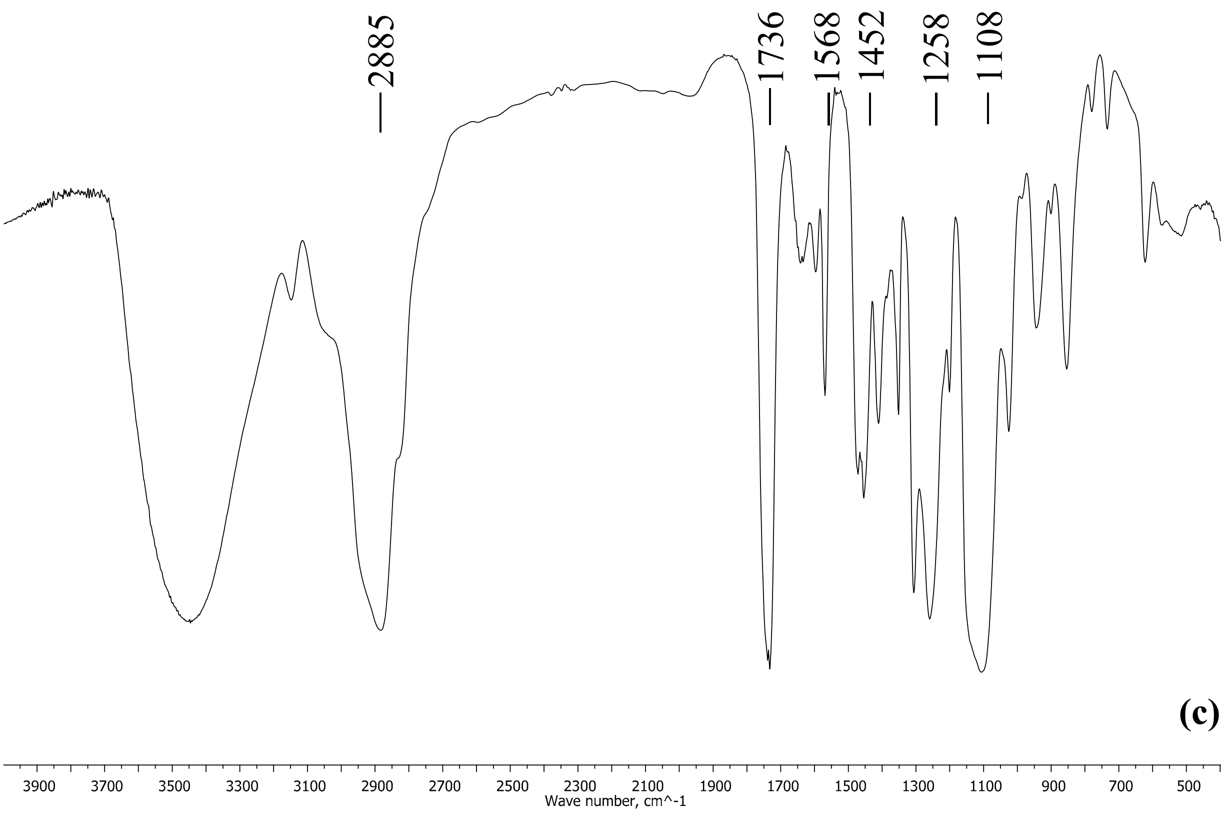


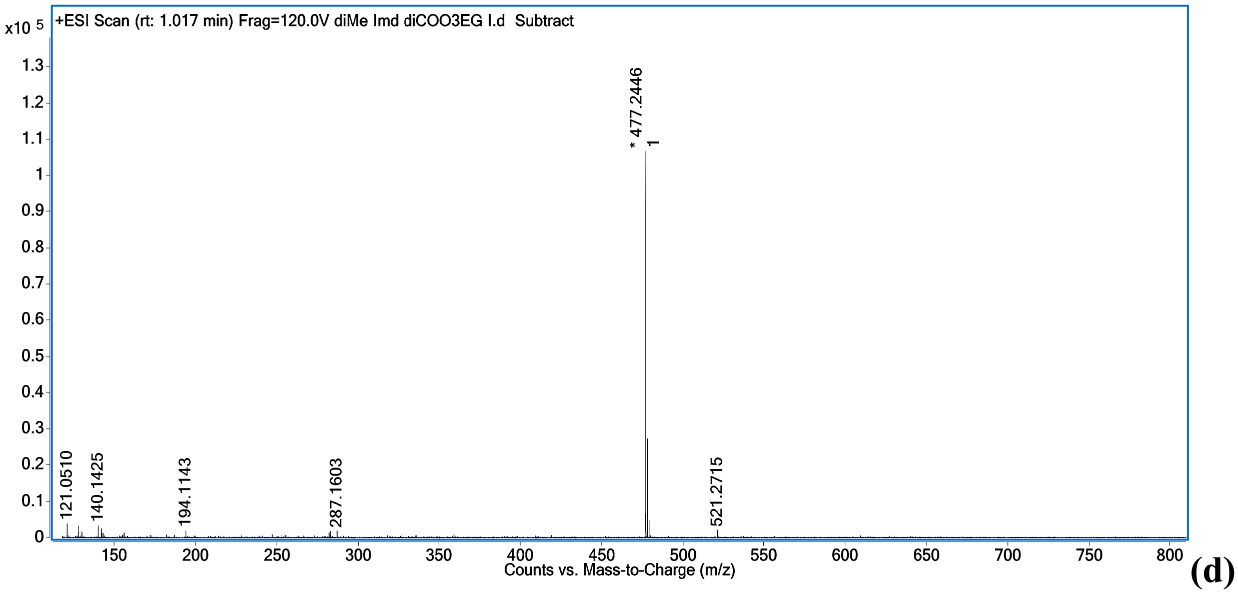


**Figure S4.** NMR $^{1}H$ (a), $^{13}C$ (b), FT IR (c) and HRESI MS (d) spectra of 4,5-bis[2-(2-(2-methoxyethoxy)ethoxy)ethoxycarbonyl]-1,3-dimethyl-imidazolium iodide (**3a**)

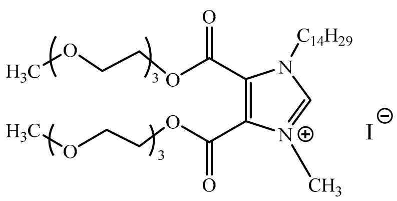
$C_{14}H_{29}$
$H_3C$
O
3
N
$N^{\oplus}$
$CH_3$
$I^{\ominus}$

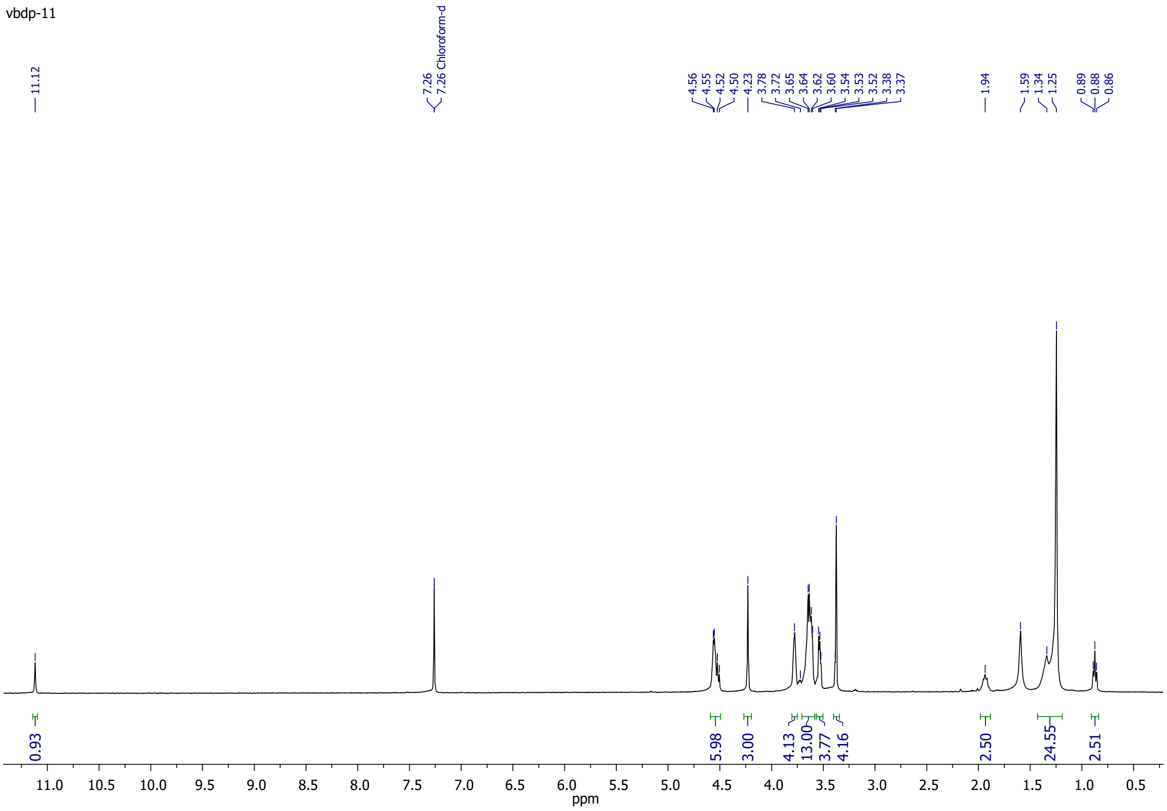
vbdp-11
11.12
7.26
7.26 Chloroform-d
4.56
4.55
4.52
4.50
4.23
3.78
3.72
3.65
3.64
3.62
3.60
3.54
3.53
3.52
3.38
3.37
1.94
1.59
1.34
1.25
0.89
0.88
0.86
0.93
5.98
3.00
4.13
13.00
3.77
4.16
2.50
24.55
2.51
ppm

(a)

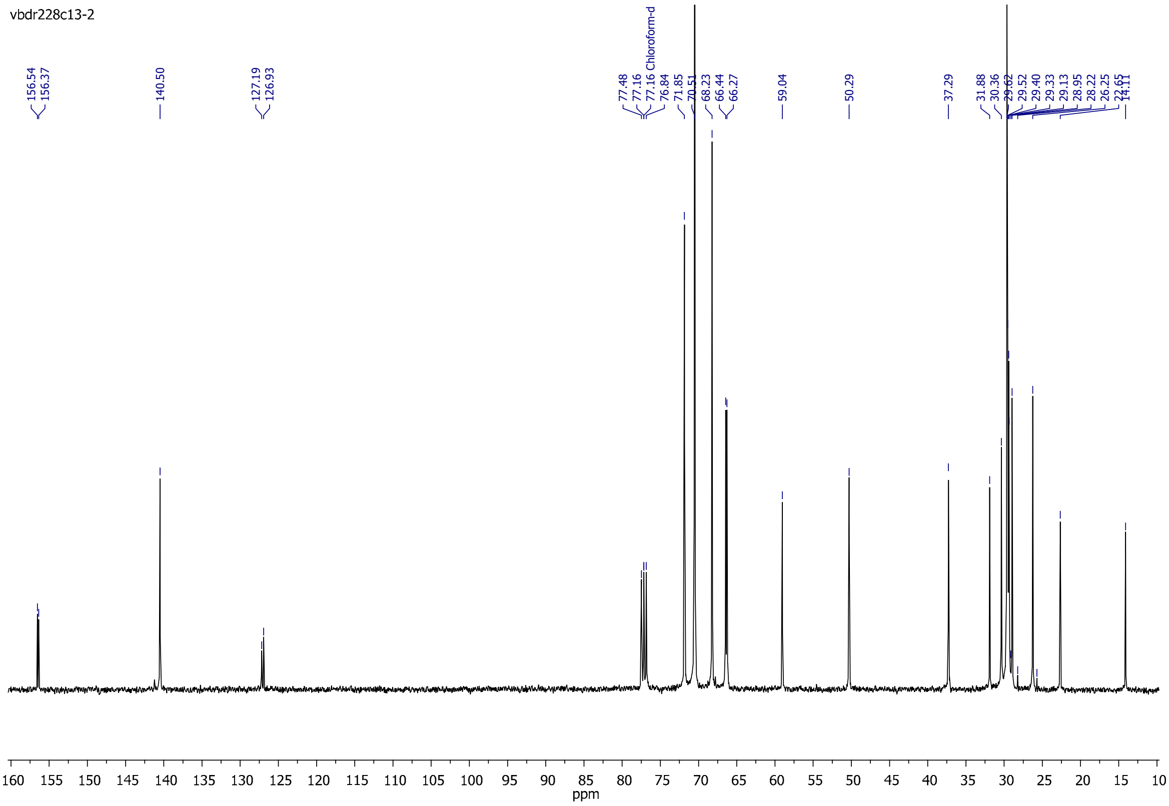
vbdr228c13-2
156.54
156.37
140.50
127.19
126.93
77.48
77.16
77.16 Chloroform-d
76.84
71.85
70.51
68.23
66.44
66.27
59.04
50.29
37.29
31.88
30.36
29.62
29.52
29.40
29.33
29.13
28.95
28.22
26.25
22.65
14.11
ppm

(b)

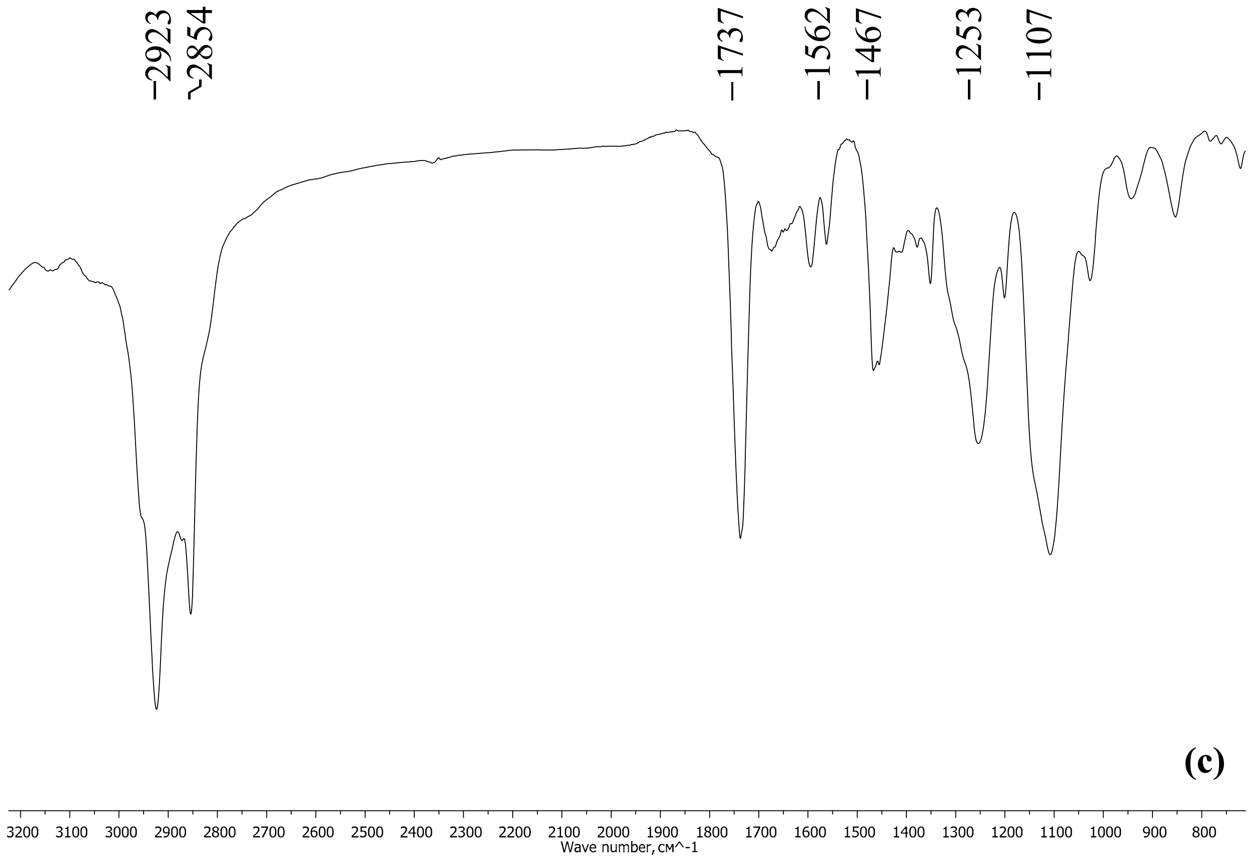


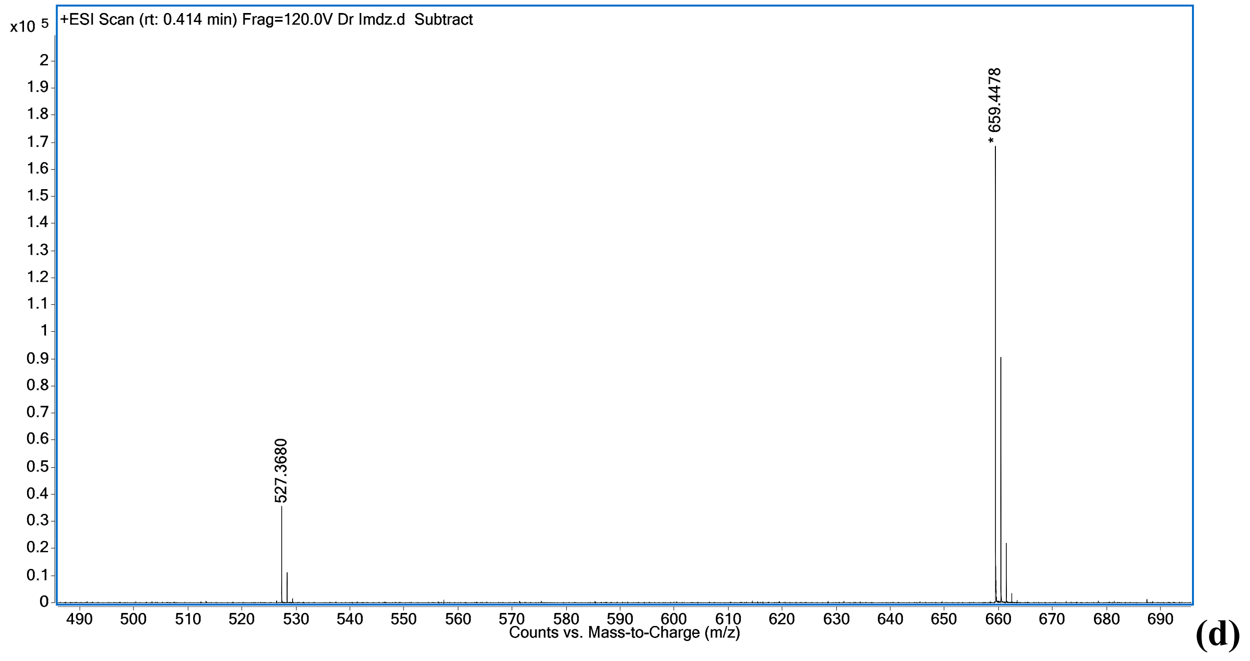


**Figure S5.** NMR $^{1}H$ (a), $^{13}C$ (b), FT IR (c) and HRESI MS (d) spectra of 4,5-bis[2-(2-(2-methoxyethoxy)ethoxy)ethoxycarbonyl]-1-methyl-3-tetradecylimidazolium iodide **(3b)**

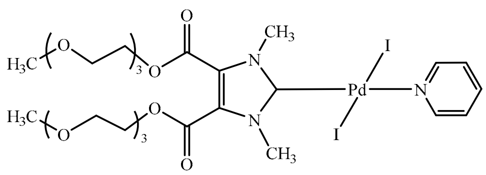

H3C
O
3
O
O
N
CH3
Pd
I
N
I
H3C
O
3
O
O
N
CH3

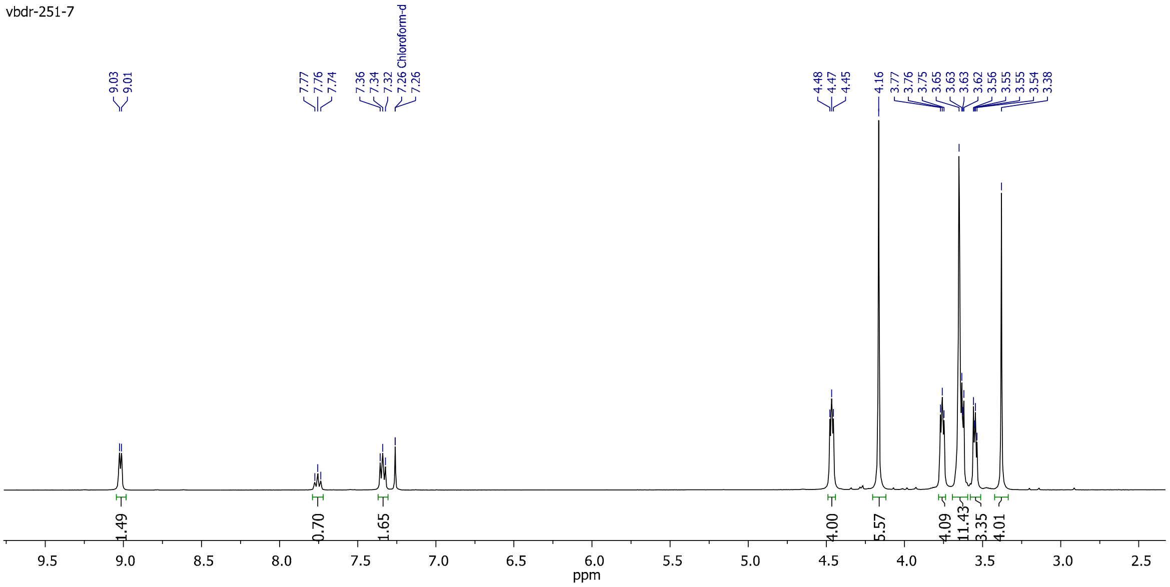

vbdr-251-7
9.03
9.01
7.77
7.76
7.74
7.36
7.34
7.32
7.26 Chloroform-d
7.26
4.48
4.47
4.45
4.16
3.77
3.76
3.75
3.65
3.63
3.63
3.62
3.56
3.55
3.55
3.54
3.38
1.49
0.70
1.65
4.00
5.57
4.09
11.43
3.35
4.01
ppm

(a)

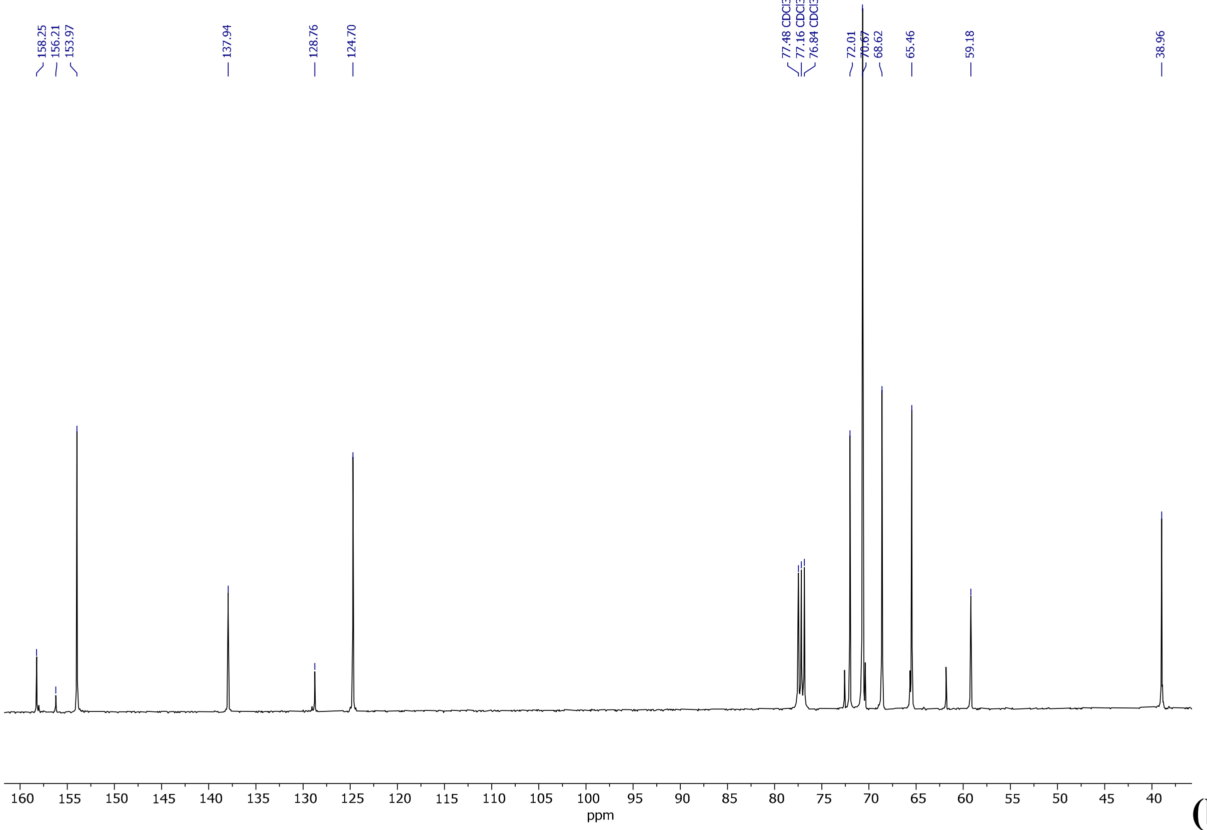

158.25
156.21
153.97
137.94
128.76
124.70
77.48 CDCl3
77.16 CDCl3
76.84 CDCl3
72.01
70.67
68.62
65.46
59.18
38.96
ppm

(b)

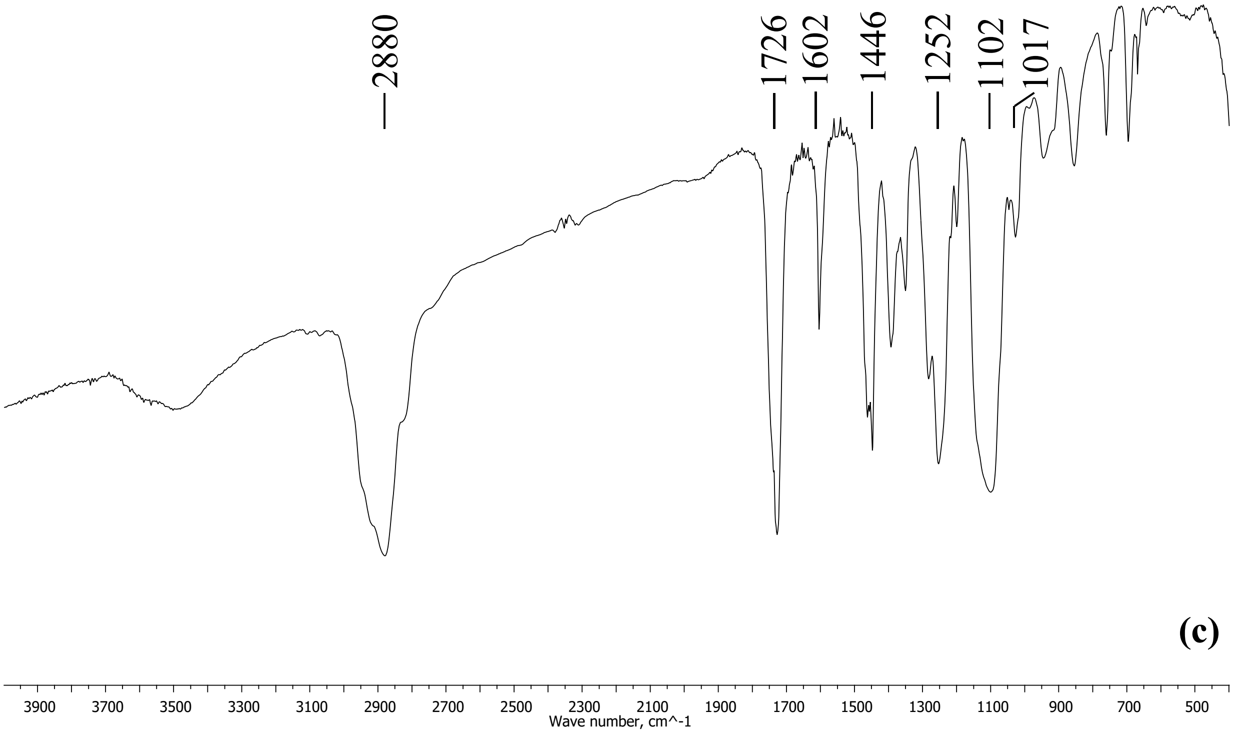


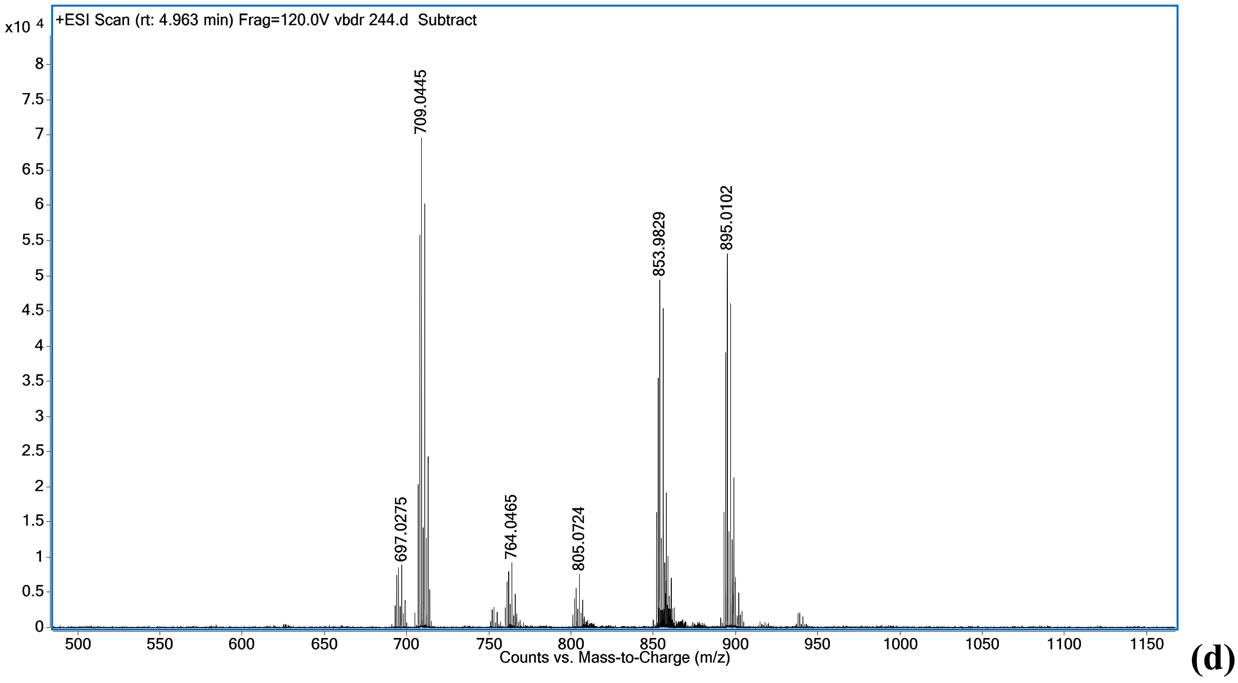


**Figure S6.** NMR $^{1}H$ (a), $^{13}C$ (b), FT IR (c) and HRESI MS (d) spectra of *trans*-{4,5-bis[2-(2-(2-methoxyethoxy)ethoxy)ethoxycarbonyl]-1,3-dimethylimidazolin-2-ylidene}{pyridine}palladium(II) diiodide **(4a)**.

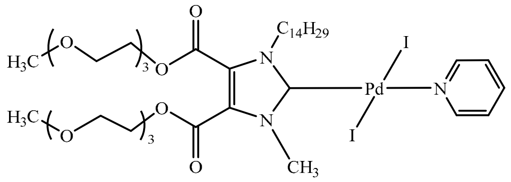

O
$C_{14}H_{29}$
I
$H_3C$
O
3
O
N
Pd
N
$H_3C$
O
O
3
N
I
O
$CH_3$


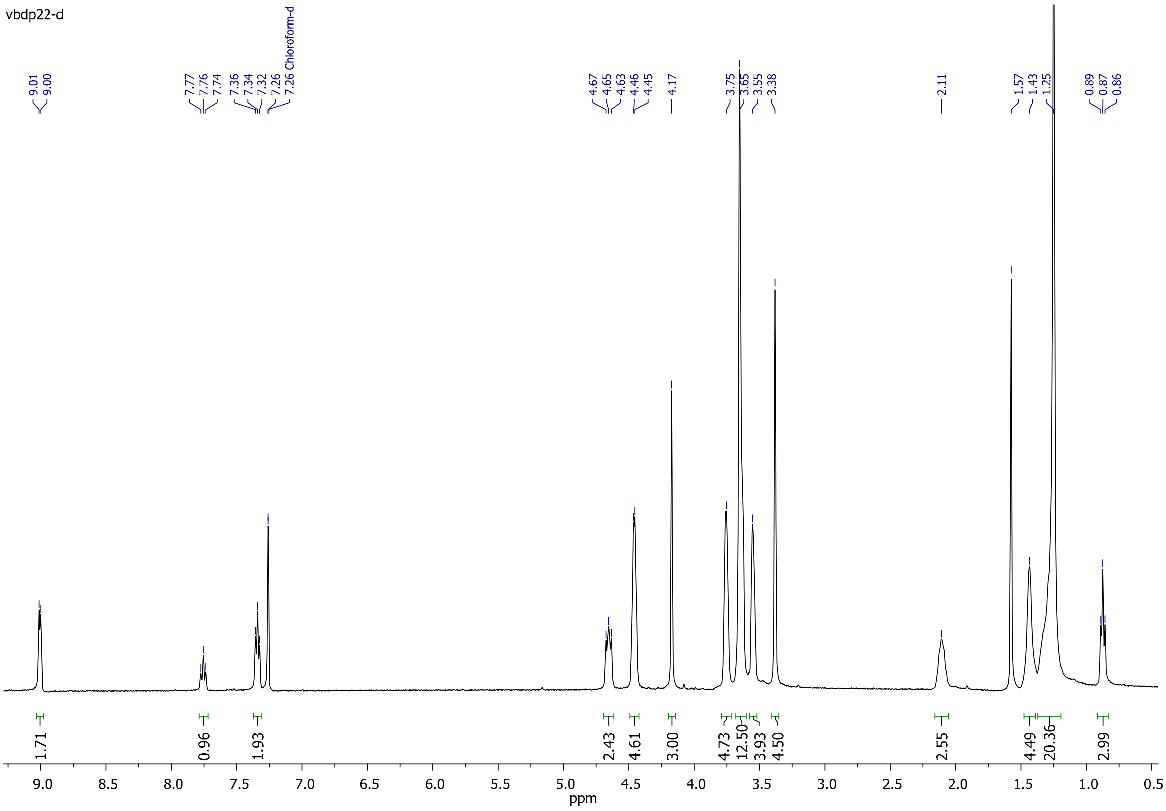

vbdp22-d
9.01
9.00
7.77
7.76
7.74
7.36
7.34
7.32
7.26
7.26 Chloroform-d
4.67
4.65
4.63
4.46
4.45
4.17
3.75
3.65
3.55
3.38
2.11
1.57
1.43
1.25
0.89
0.87
0.86
1.71
0.96
1.93
2.43
4.61
3.00
4.73
12.50
3.93
4.50
2.55
4.49
20.36
2.99
9.0 8.5 8.0 7.5 7.0 6.5 6.0 5.5 5.0 4.5 4.0 3.5 3.0 2.5 2.0 1.5 1.0 0.5
ppm


**(a)**

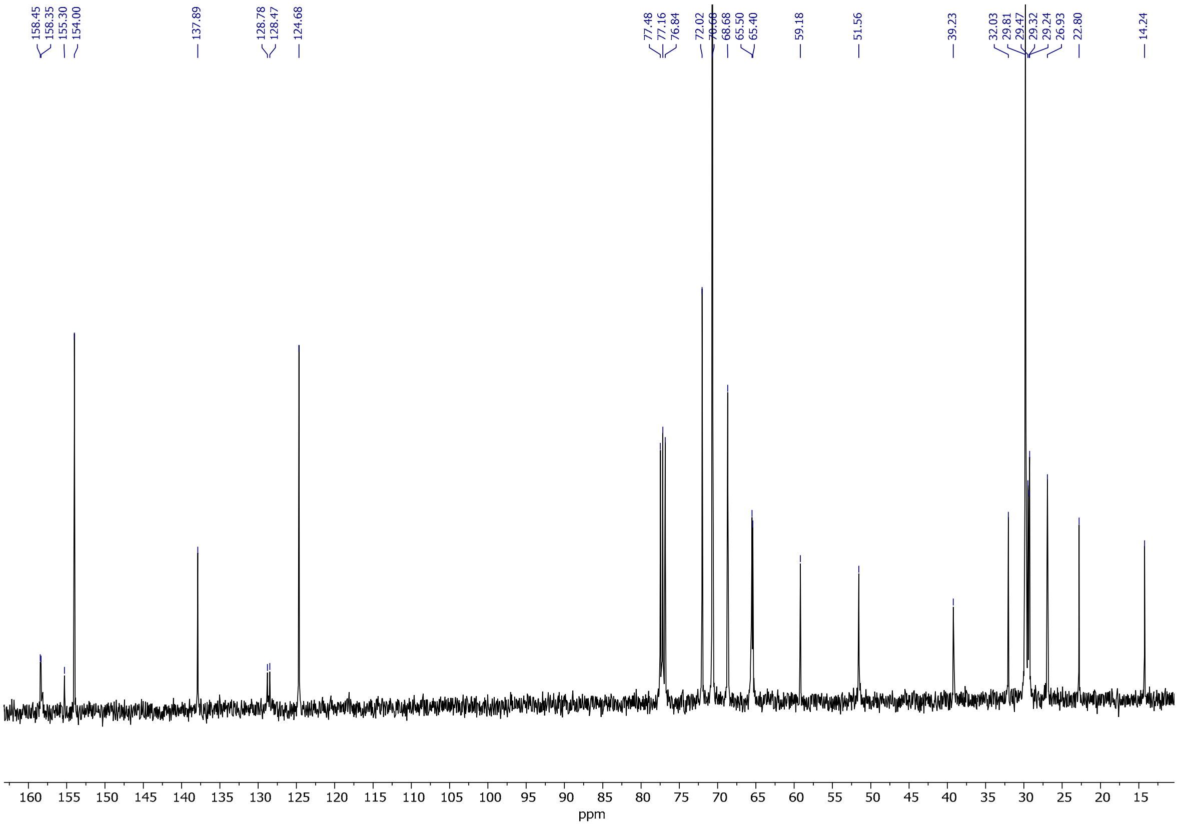

158.45
158.35
155.30
154.00
137.89
128.78
128.47
124.68
77.48
77.16
76.84
72.02
70.60
68.68
65.50
65.40
59.18
51.56
39.23
32.03
29.81
29.47
29.32
29.24
26.93
22.80
14.24
160 155 150 145 140 135 130 125 120 115 110 105 100 95 90 85 80 75 70 65 60 55 50 45 40 35 30 25 20 15
ppm


**(b)**

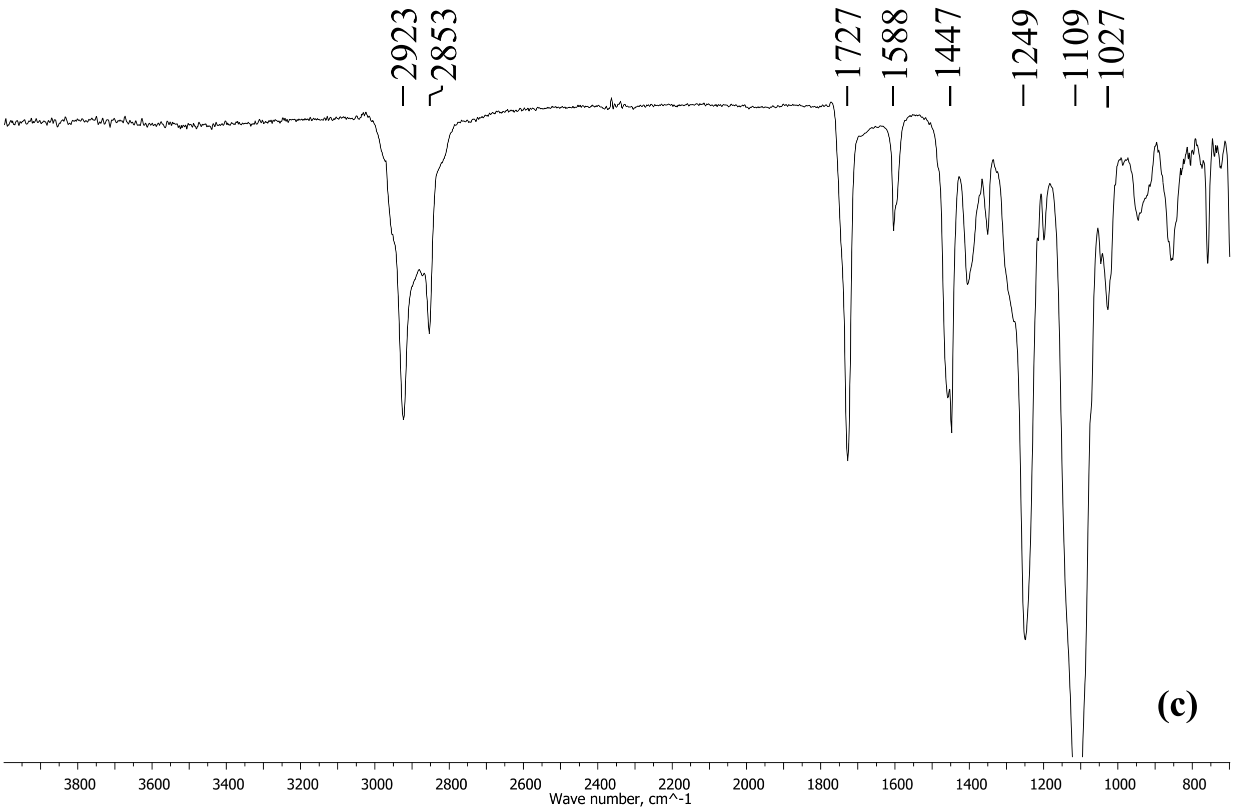


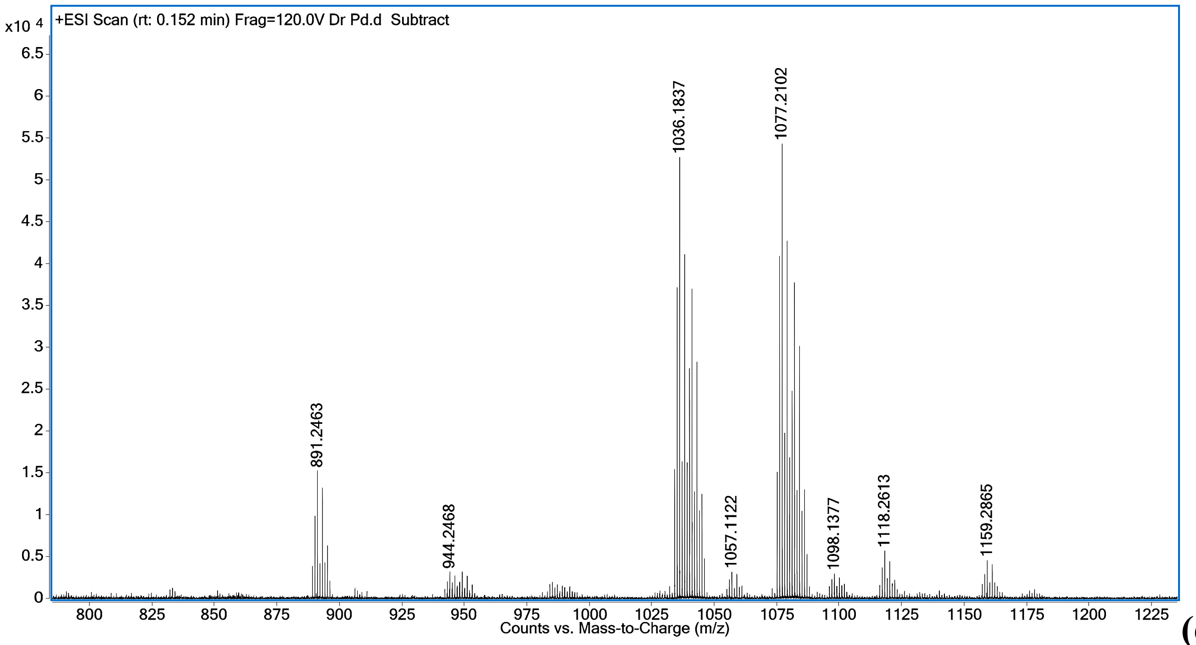


**(d)**

**Figure S7.** NMR $^{1}H$ (a), $^{13}C$ (b), FT IR (c), HRESI MS (d) spectra of *trans*-{4,5-bis[2-(2-(2-methoxyethoxy)ethoxy)ethoxycarbonyl]-1-methyl-3-tetradecylimidazolin-2-ylidene}{pyridine}palladium(II) diiodide **(4b)**.

| | | | |
|---|---|---|---|
| Temperature (°C): | 25,0 | Duration Used (s): | 60 |
| Count Rate (kcps): | 347,4 | Measurement Position (mm): | 1,05 |
| Cell Description: | Disposable sizing cuvette | Attenuator: | 10 |

| | | | Size (d.nm): | % Intensity: | St Dev (d.nm): |
|---|---|---|---|---|---|
| Z-Average (d.nm): | 254,0 | Peak 1: | 145,2 | 45,6 | 56,59 |
| PdI: | 0,637 | Peak 2: | 499,8 | 37,6 | 215,2 |
| Intercept: | 0,787 | Peak 3: | 4456 | 16,8 | 905,0 |
| Result quality : | Good | | | | |

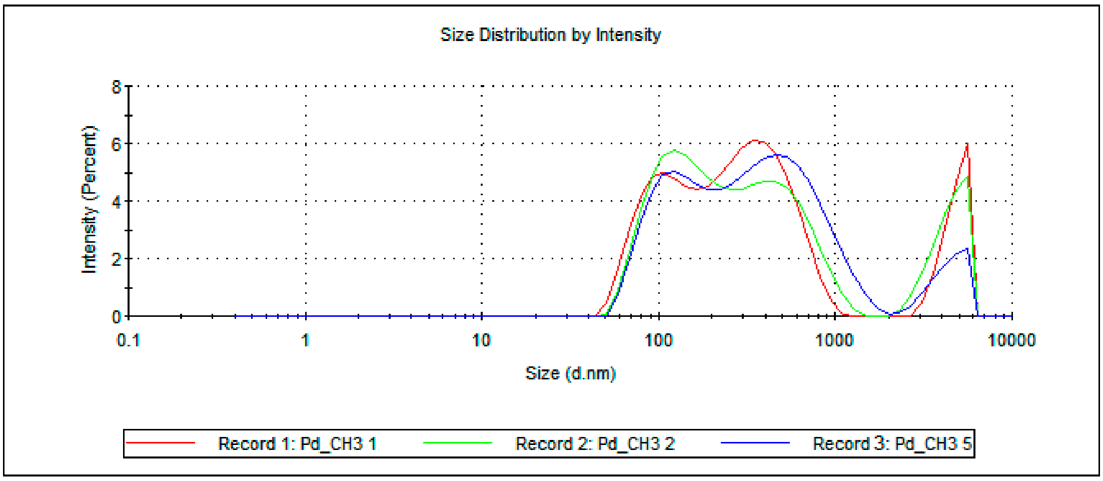


(A)

| | | | |
|---|---|---|---|
| Temperature (°C): | 25,0 | Duration Used (s): | 60 |
| Count Rate (kcps): | 293,3 | Measurement Position (mm): | 4,65 |
| Cell Description: | Disposable sizing cuvette | Attenuator: | 7 |

| | | | Size (d.nm): | % Intensity: | St Dev (d.nm): |
|---|---|---|---|---|---|
| Z-Average (d.nm): | 177,7 | Peak 1: | 194,0 | 100,0 | 60,15 |
| PdI: | 0,112 | Peak 2: | 0,000 | 0,0 | 0,000 |
| Intercept: | 0,950 | Peak 3: | 0,000 | 0,0 | 0,000 |
| Result quality : | Good | | | | |

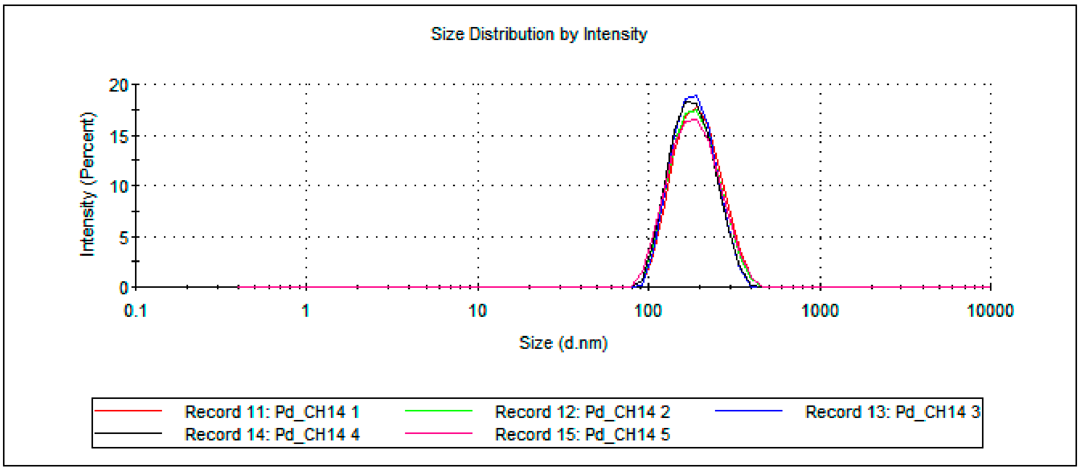


(B)

**Figure S8.** DLS intensity *vs* size graphs for solutions of **4a** (A) and **4b** (B) in water, C [**4a**,**b**]= 0.02 mM.
